# Taphonomy of marine vertebrates of the Pisco Formation (Miocene, Peru): Insights into the origin of an outstanding Fossil-Lagerstätte

**DOI:** 10.1371/journal.pone.0254395

**Published:** 2021-07-15

**Authors:** Giulia Bosio, Alberto Collareta, Claudio Di Celma, Olivier Lambert, Felix G. Marx, Christian de Muizon, Anna Gioncada, Karen Gariboldi, Elisa Malinverno, Rafael Varas Malca, Mario Urbina, Giovanni Bianucci

**Affiliations:** 1 Dipartimento di Scienze dell’Ambiente e della Terra, Università degli Studi di Milano-Bicocca, Milan, Italy; 2 Dipartimento di Scienze della Terra, Università di Pisa, Pisa, Italy; 3 Scuola di Scienze e Tecnologie, Università di Camerino, Camerino, Italy; 4 D.O. Terre et Histoire de la Vie, Institut Royal des Sciences Naturelles de Belgique, Bruxelles, Belgium; 5 Museum of New Zealand Te Papa Tongarewa, Wellington, New Zealand; 6 Department of Geology, University of Otago, Dunedin, New Zealand; 7 Département Origines et Evolution, CR2P UMR 7207, (MNHN, CNRS, UPMC, Sorbonne-Université), Muséum national d’Histoire naturelle, Paris, France; 8 Departamento de Paleontologia de Vertebrados, Museo de Historia Natural-UNMSM, Lima, Peru; Ecole normale superieure de Lyon, FRANCE

## Abstract

The Miocene Pisco Formation, broadly exposed in the Ica Desert of southern Peru, is among the most outstanding Cenozoic marine Fossil-Lagerstätten worldwide. It is renowned for its exceptional preservation and abundance of vertebrate fossils, including a rich assemblage of whales and dolphins (Cetacea). Here, we integrate taphonomic data on 890 marine vertebrate fossils, gathered through 16 different localities. Our observations range from the taxonomic distribution, articulation, completeness, disposition and orientation of skeletons, to the presence of bite marks, associations with shark teeth and macro-invertebrates, bone and soft tissue preservation, and the formation of attendant carbonate concretions and sedimentary structures. We propose that the exceptional preservation characterising many Pisco vertebrates, as well as their exceptionally high abundance, cannot be ascribed to a single cause like high sedimentation rates (as proposed in the past), but rather to the interplay of several favourable factors including: (i) low levels of dissolved oxygen at the seafloor (with the intervention of seasonal anoxic events); (ii) the early onset of mineralisation processes like apatite dissolution/recrystallisation and carbonate mineral precipitation; (iii) rapid burial of carcasses in a soupy substrate and/or a novel mechanism involving scour-induced self-burial; and (iv) original biological richness. Collectively, our observations provide a comprehensive overview of the taphonomic processes that shaped one of South America’s most important fossil deposits, and suggest a model for the formation of other marine vertebrate Fossil-Lagerstätten.

## Introduction

Fossils of Cenozoic marine vertebrates have prompted taphonomic research and speculation since the Renaissance [1]. Recent studies have explored many aspects of their preservation, including the biostratinomic signature of ancient mass strandings [2], the origin of bonebeds [3], the factors controlling taphonomic gradients across onshore-offshore transects [4], the impact of habitat preferences [5] and sea-level changes [6] on vertebrate preservation, the trace and body fossils of vertebrate [7] and invertebrate [8] scavengers, and the onset of complex whale-fall communities [9].

The Pisco Formation, exposed in the Ica Desert of southern coastal Peru, is a globally significant fossil deposit known for its outstanding assemblage of Miocene sharks and rays, bony fishes, marine turtles and crocodiles, seabirds, cetaceans, and pinnipeds [10–21]. Four decades of research on these specimens have unveiled an unusual quality and quantity of palaeontological information, thus qualifying the Pisco Formation as a Fossil-Lagerstätte (as per the original definition by Seilacher [22]) [14, 23, 24]. In particular, exceptionally preserved soft tissues [12–14, 25, 26], bone proteins [27], and digestive tract contents [28–30] imply the existence of a true Konservat-Lagerstätte (*sensu* Seilacher [22] and Allison [31]). Combined with extensive outcrops, numerous fossil localities, a wide stratigraphic range, and its connection with one of the world’s most productive marine ecosystems (the present-day Humboldt Current; [32]), these characteristics make the Pisco Formation a prime opportunity to unravel the patterns and processes driving marine vertebrate fossilisation.

Previous analyses of the taphonomic history of the Pisco assemblage focused mainly on baleen whales, and provided relatively little systematic, chronological, or palaeoenvironmental detail on the investigated specimens [12–14, 33, 34]. More recently, a multidisciplinary team of palaeontologists, stratigraphers, sedimentologists, micropalaeontologists, and mineral scientists conducted a multi-year investigation of the taphonomic processes at play during the deposition of the Pisco Formation. Results from this programme include detailed censuses of key localities [17, 18, 35], their contextualisation into a comprehensive chronostratigraphic and palaeoenvironmental framework [19, 36–38], detailed investigations of exceptionally preserved specimens [25, 26, 28–30], and preliminary insights into early diagenetic processes [23, 24, 39, 40].

Here, we provide a synoptic overview and new interpretation of published and unpublished taphonomic data on 890 vertebrate fossils, gathered during 15 field campaigns involving 16 different localities. By synthesising insights from a variety of disciplines, we propose a new model for the formation of this exceptionally rich deposit, which in turn may help to elucidate the preservational mechanisms behind Fossil-Lagerstätten elsewhere.

## Geological and stratigraphic settings

**Fig 1:**
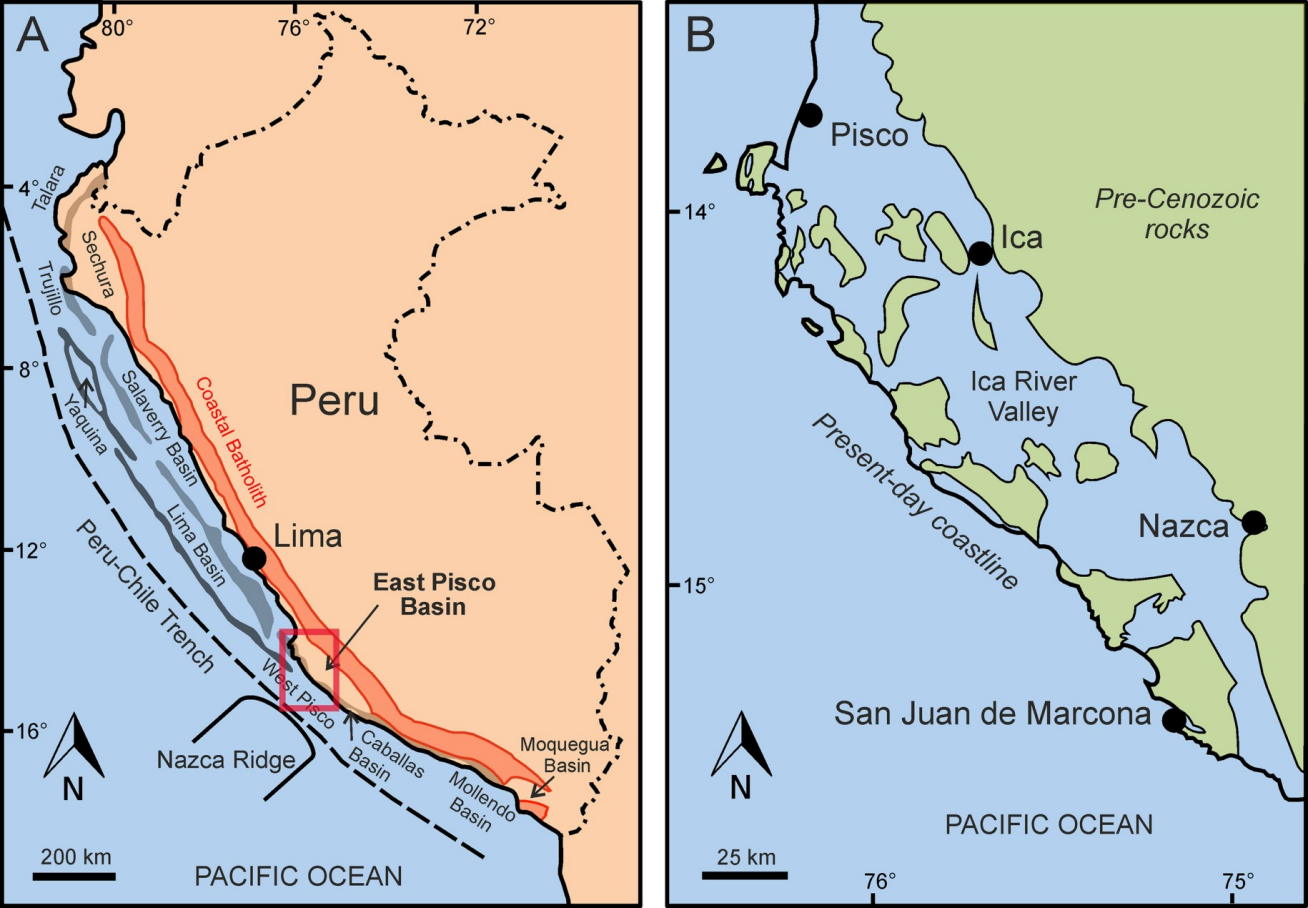
Geographic and palaeogeographic setting of the study area. A) Map of the Peruvian coast showing the distribution of the main Cenozoic forearc sedimentary basins. Major structural highs are the Coastal Batholith (red line), the Outer Shelf High and the Upper Slope Ridge (dark grey- and black-shaded areas, respectively). B) Palaeogeographic reconstruction of the Miocene Pisco embayment along the Peruvian coast, based on the distribution of the pre-Cenozoic rocks. Redrawn and modified from Bosio et al. ([35]: Fig 1) and references therein.

The Peruvian forearc system comprises an inner set of shelf basins and a seaward set of slope basins separated by a prominent, trench-parallel structural ridge, the Outer Shelf High [41]. An additional structural ridge, the Upper-Slope Ridge, bounds the offshore outer basins on their southwestern side (Fig 1).

The East Pisco Basin (EPB) is one of these forearc shelf basins and contains middle Eocene to Pliocene deposits along the southern sector of the Peruvian convergent margin [42]. The subaerial portion of this elongate, trench‐parallel basin extends for about 180 km along a narrow coastal plain between the cities of Pisco and Nazca; it is separated from the offshore West Pisco Basin to the southwest by the Coastal Cordillera, an emerging basement high composed of Precambrian and Palaeozoic metamorphic and igneous rocks representing the southern and onshore prolongation of the Outer Shelf High [41, 43].

The sedimentary infill of the EPB exceeds 1,000 m in thickness and primarily comprises marine mudstones, siltstones, diatomites and diatomaceous siltstones, with minor intercalations of sandstones, conglomerates, and volcanic ash layers. The basin succession rests on a complex pre-Cenozoic basement consisting of a core of Precambrian metamorphic rocks, known as the Arequipa Massif [44–46]. The latter was intruded by an assemblage of lower Palaeozoic gabbroic to granitoid rocks forming the San Nicolás Batholith [47] that, in turn, is unconformably overlain by Jurassic volcano-sedimentary rocks [48].

In the study area, the basin filling sedimentary succession comprises four main lithostratigraphic units including, from bottom to top, the Paracas Formation (composed of the Los Choros and Yumaque members), the Otuma Formation, the Chilcatay Formation, and the Pisco Formation [49–51]. These units are bounded by regionally extensive, conglomerate-mantled unconformities that are locally accompanied by angular discordances.

**Fig 2:**
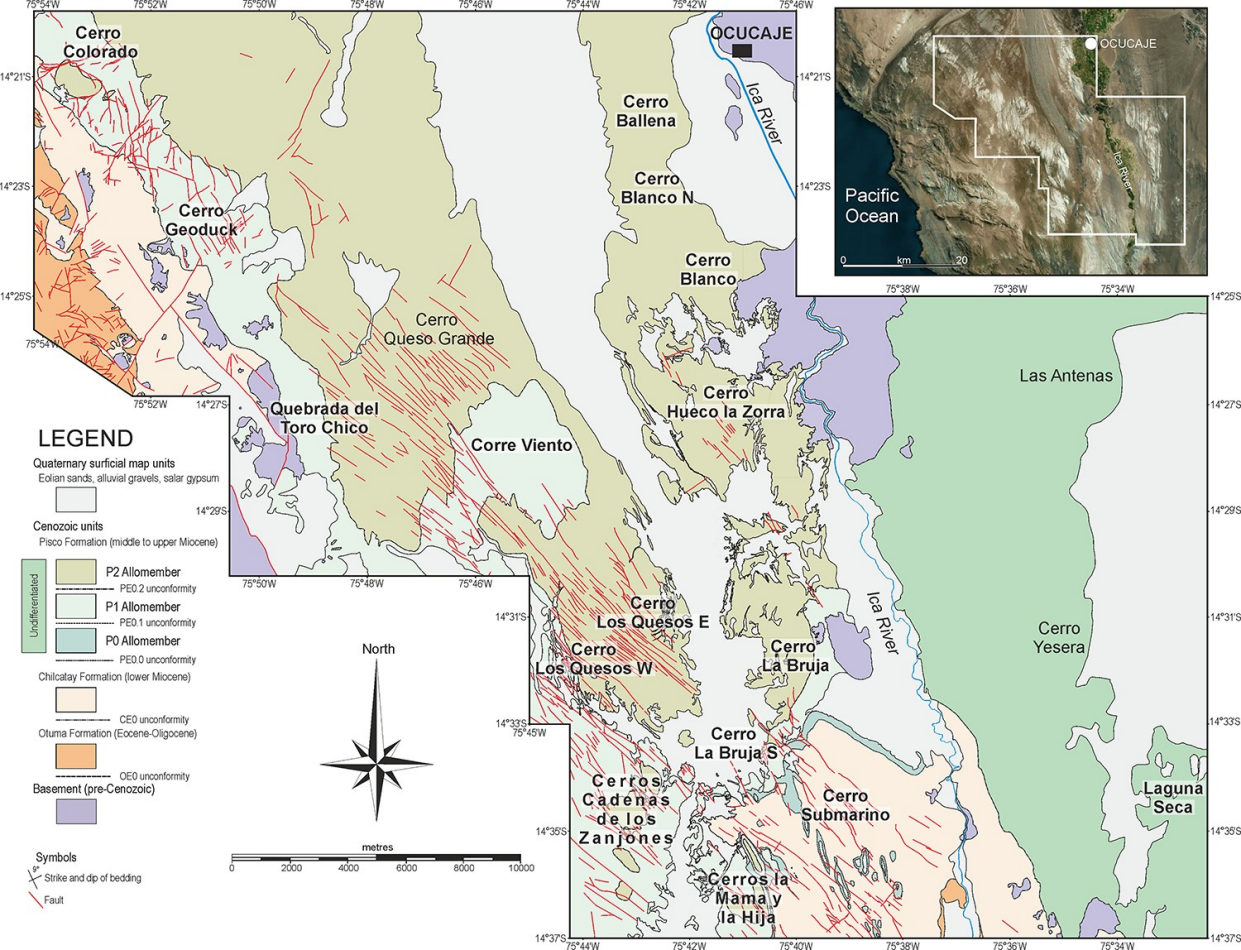
Geological map. Simplified geological map of the investigated exposures of the Pisco Formation in the Ocucaje area, including all the localities that are mentioned in the present work (see Fig 4 for stratigraphic sections showing the position of the prospected fossils). The inset satellite image was obtained under open license from Google Earth. Locality names highlighted in bold indicate sixteen fossil-rich sites where thorough surveys for marine vertebrates have been performed (see S1 Table).

Extensive field mapping and sedimentological study of outcrop sections along the Ica River [19, 52] have shown the base of the Pisco Formation (PE0) to be a diachronous composite surface resulting from the landward (i.e. northeastward) convergence of at least three regionally mappable unconformities, named PE0.0 through PE0.2 from oldest to youngest (Fig 2). Each of these unconformities reflects a separate sea-level encroachment. Consequently, the Pisco Formation is a cyclical sediment unit composed of at least three depositional sequences (or allomembers), designated P0, P1, and P2 in stratigraphic order. Internally, each sequence preserves a record of water-depth change and displays an overall fining-upward trend of facies associations documenting a gradual vertical shift from shoreface to offshore deposition. Integration of biostratigraphic and tephrochronological age determinations with Strontium Isotope Stratigraphy constrains the ages of P0, P1, and P2 to 14.8–12.4 Ma, 9.5–8.6 Ma, and 8.4–6.7 Ma, respectively [36–38].

## Data and research methods

### Data compilation

The Pisco Formation crops out extensively in the vicinity of Ocucaje (Ica Province) along the western and eastern sides of the lower Ica Valley. This desert region is characterised by the presence of several hills (locally known as “cerros”) and preserves an exceptionally high concentration of fossil vertebrates. Starting in 2006, and for some 15 field seasons, our multidisciplinary team focused on establishing a detailed census of the fossil vertebrates cropping out at several fossil-rich exposures of the Pisco Formation. Preliminary results of this long-term research effort included the production of detailed thematic maps displaying the spatial and stratigraphic distribution of hundreds of fossil vertebrates from the key localities of Cerro Colorado, Cerro Los Quesos, and a broad area in the vicinity of Cerro Submarino [17, 18, 35]. Here, we apply a similar approach to a total of 16 localities (Fig 2), including the aforementioned three, which together involves first-hand observations on 890 vertebrate fossils. The area surveyed at each site varies depending on the local circumstances (see details below).

**Fig 3:**
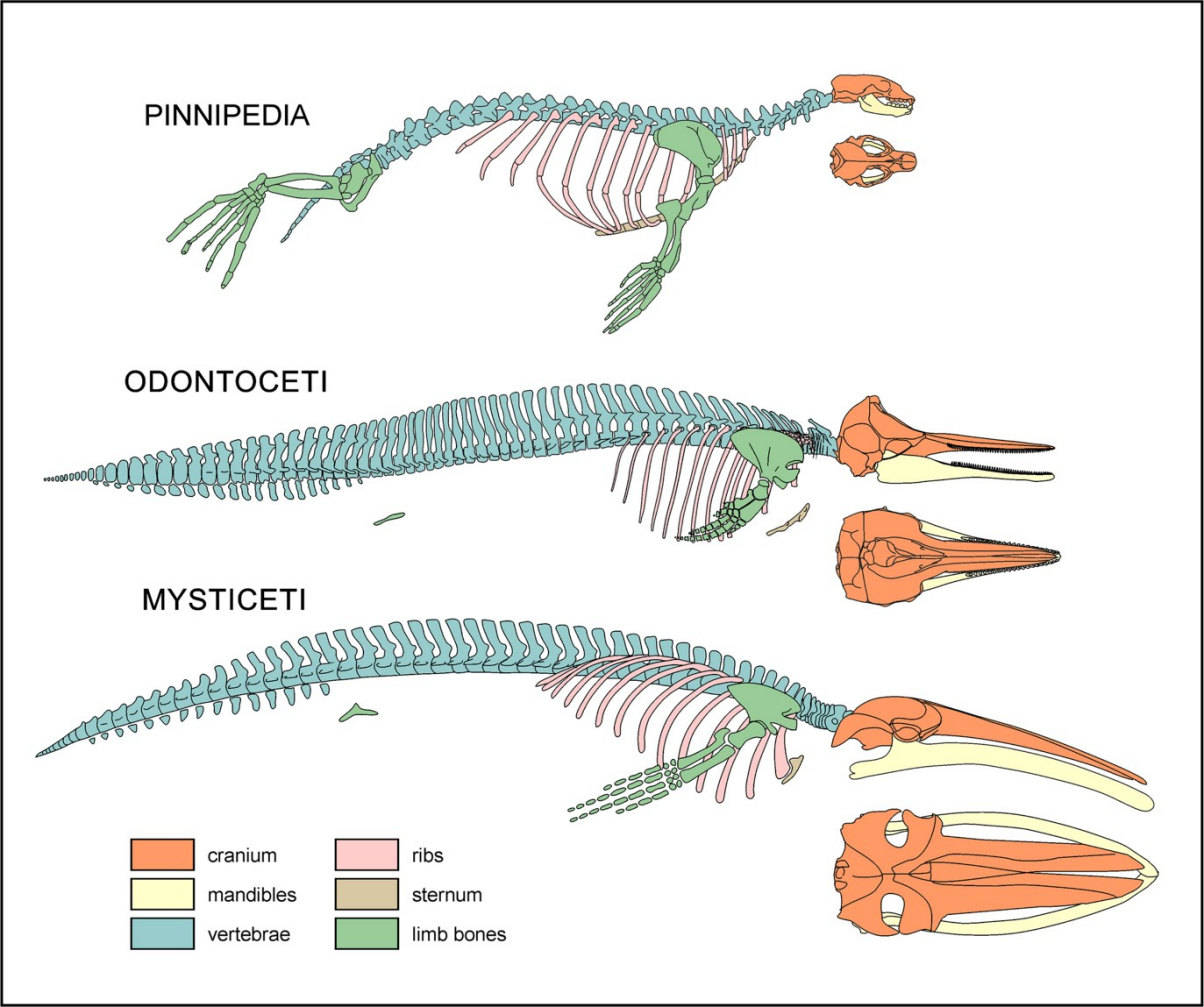
Simplified anatomical sketches. Schematic skeletons of a seal (Carnivora: Pinnipedia), a toothed whale (Cetacea: Odontoceti) and a baleen whale (Cetacea: Mysticeti) showing the main anatomical districts (cranium, mandibles, vertebrae, ribs, sternum and limb bones) taken into considerations in the present work. Complete skeletons are depicted in lateral view; additional sketches of the crania in dorsal view are also provided.

We scanned each locality for vertebrate fossils via systematic surface prospecting, making sure not to overestimate specimen counts based on dislocated bones. The exact position of each fossil was recorded using hand-held global positioning system (GPS) receivers. Taphonomic observations and preliminary identifications were performed directly in the field, with reference to the main osteoanatomical regions illustrated in Fig 3. Descriptions were frequently hampered by bone weathering (especially along steep slopes and windswept flatlands) and specimens remaining partially buried in the rock. To facilitate data collection, several fossils were partially excavated, but then covered again with loose sediment to slow further chemical and mechanical deterioration. Particularly significant finds were collected and deposited at the Museo de Historia Natural de la Universidad Nacional Mayor de San Marcos (= MUSM) in Lima, Peru.

For each specimen, the degree of skeletal articulation was recorded as follows: 4, 100–75% of the bones articulated; 3, 75–50%; 2, 50–25%; 1, <25%; 0, bones are fully disarticulated. Similarly, for skeletal completeness we used: 4, 100–75% of the skeleton preserved; 3, 75–50%; 2, 50–25%; 1, < 25%; 0, isolated bone or compound skeletal element (e.g. the cranium). Degrees of articulation and completeness were summarised via bivariate bubble plots in Microsoft Excel [53–56]. Where applicable, we also noted the disposition of the specimen (e.g. dorsal, ventral or lateral side up), its orientation with respect to geographic north, the presence of bite marks and/or associated faunal elements, the presence and development of an associated concretion, the extent of recent erosion, and sedimentary structures related to the burial phase. For cetaceans, which are abundant and often relatively complete, the following observations were also taken when possible: total length of the skeleton, condylobasal length and bizygomatic width of the cranium (useful for estimating total body length [15, 57]), and degree of fusion of the vertebral epiphyses (a proxy for physical maturity [58, 59]).

**Fig 4:**
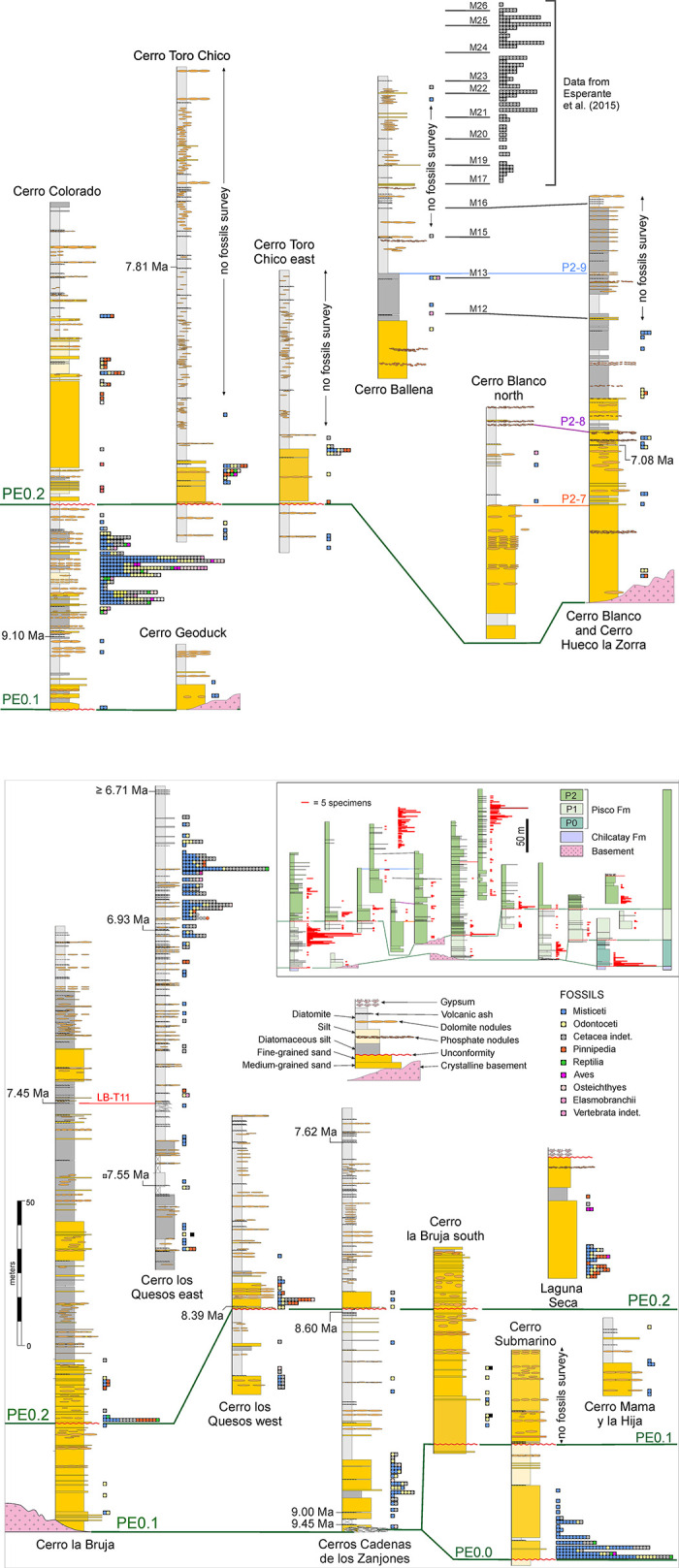
Stratigraphic sections. Stratigraphic sections (in metres) of sixteen localities of the Pisco Formation in the Ocucaje area, showing the distribution of the prospected fossil vertebrates (see Fig 2 for a geological map including the geographic distribution of these localities). The position of marker beds, intraformational unconformities, and radiometric (^40^Ar/^39^Ar) ages for dated volcanic ash layers are also indicated. Marker beds and dated ash layers after Bosio et al. [38].

Several detailed sedimentary logs, here presented as fifteen composite stratigraphic columns (Fig 4), were measured at the decimetre scale for the sixteen study localities using a Jacob’s staff, taking into account the bedding attitude in terms of strike and dip. All of the encountered fossils were then positioned along these stratigraphic sections with an accuracy of ±0.4 to ±3.0 m.

As a whole, these efforts produced a large and detailed fossil vertebrate database that is provided as a series of spreadsheets in the S1 Table.

### Analytical methods

For our taphonomic analyses, we prepared thin sections from small fragments of several cetacean specimens (some selected bones–mostly ribs–and baleen) and the entombing carbonate concretions at the Università di Pisa. Sampling used the smallest amount of material necessary, avoiding bones with evidence of transport and/or reworking. Bone fragments were embedded in epoxy resin, cut with a diamond saw, and then polished with silicon carbide and alumina. They were then examined using both transmitted and reflected light under an optical microscope Zeiss Axioplan. In addition, thin sections and polished bone fragments were analysed via scanning electron microscopy (SEM) with a Quanta 450 FESEM and a Philips XL30 SEM equipped with a DX4i EDAX microanalyser. The SEM-EDS analytical conditions were 20 kV accelerating voltage, 5 nA beam current and 10 mm working distance.

Further details regarding the analytical methods (e.g. EPMA, XRD) of taphonomic investigations are available elsewhere [23, 25, 26, 38–40, 56].

## Taphonomy

### Taxonomic distribution

**Fig 5:**
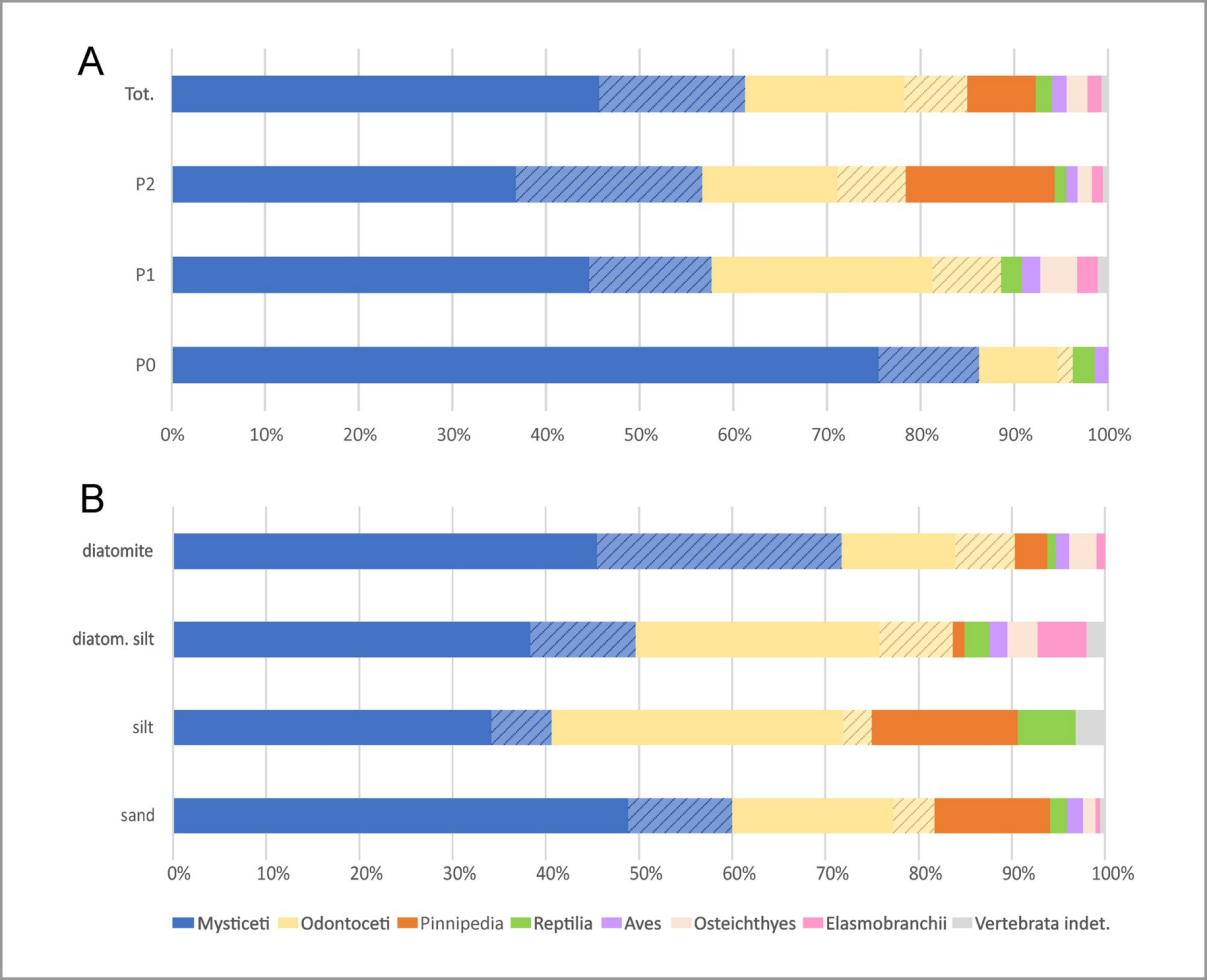
Taxonomic distribution. Bar diagrams showing the distribution of the taxonomic groups of marine vertebrates through the three sequences (A) and main lithologies (B) of the Pisco Formation in the study area. Note that the indeterminate cetacean specimens (highlighted by lined patterns in the diagrams below) have here been redivided into mysticetes and odontocetes by following the relative proportions in which these groups are observed in each sequence and lithology.

Cetaceans dominate all of the 16 prospected localities (Fig 4), representing about 85% of our total of 890 specimens. Baleen whales (mysticetes) are significantly more abundant (ca 61%) than toothed whales (odontocetes, ca 24%) (Fig 5A). Of the remaining specimens, ca 7% represent pinnipeds, 1.8% reptiles, 1.6% birds, 2.2% bony fishes, and 1.5% elasmobranchs. These proportions notably differ from those of Esperante et al. [14] who, in approximately the same areas examined by us, identified 571 vertebrates, 98.6% of which were mysticetes, 0.7% odontocetes, and 0.7% pinnipeds.

The composition of the vertebrate assemblage varies across the three sequences of the Pisco Formation (Fig 5A). Mysticetes are relatively more abundant in P0, odontocetes more abundant in P1, and pinnipeds are only present in P2. The high percentage of mysticetes in P0 contrasts markedly with their virtual absence in the underlying Chilcatay strata [56, 60] and entirely derives from the marginal marine sands at the base of that sequence [35]. Rather than habitat preference, we suggest that this pattern might reflect selective (e.g. taxon- or size-related) transport of floating baleen whale carcasses into the sheltered P0 palaeoenvironment (see Articulation and completeness). The abundance of odontocetes in P1, which also correlates with a peak in their local diversity and disparity [19], could be due to a combination of local (i.e. palaeoenvironmental) factors and high levels of global diversity during the early late Miocene [61]. The appearance of pinnipeds in P2 represents some of the earliest evidence for the presence of this clade in the South Pacific [62].

In terms of lithology (Fig 5B), mysticete abundance decreases from diatomites to diatomaceous silts to silts, which is accompanied by a parallel increase in the abundance of odontocetes. This pattern suggests different environmental preferences for baleen and toothed whales, with the former perhaps avoiding shallow coastal waters (owing to their larger size) and/or favouring nutrient-rich areas. The inversion of this trend in the proximal shoreface sand deposits might reflect the role of marginal-marine environments as *cul-de-sacs* on which large carcasses like those of baleen whales strand and accumulate [63]. Pinnipeds are mostly found in the sandy-silty realm, testifying to their amphibious habits. Reptiles also seem to be scarce in the diatomites, whereas seabirds do not show any obvious pattern. Overall, variations in the composition of the vertebrate assemblage seem to be mostly controlled by taxon-specific environmental preferences, although taphonomic processes like post-mortem transport may also have played a role.

### Articulation and completeness

**Fig 6:**
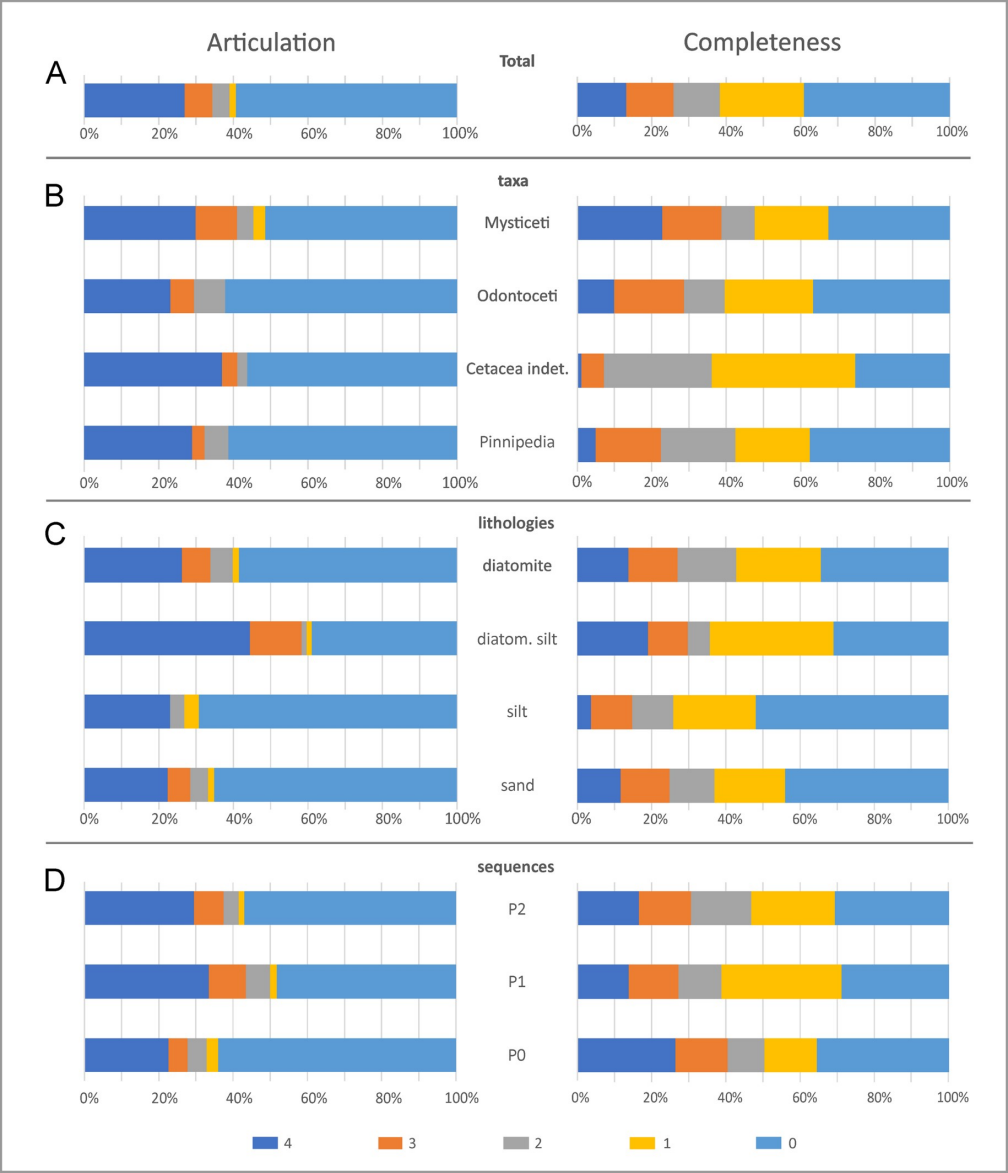
Overview of the articulation and completeness degrees. Bar diagrams showing the articulation (left column) and completeness (right column) degree distribution of the fossil marine vertebrates from the Pisco Formation with respect to various samples: the total vertebrate assemblage (A), the main taxonomic groups of marine mammals (B), the different entombing lithologies (C), and the different host sequences (D). The percentages refer to the total number of specimens.

Most of the investigated specimens (ca 59%) are fully disarticulated, i.e. they belong to the articulation class 0, which also includes isolated crania. About 27% are fully articulated, i.e. they belong to class 4, which includes many specimens consisting of a cranium with articulated mandibles. Together, classes 0 and 4 account for about 86% of the total number of specimens, resulting in a seemingly bimodal distribution (Fig 6A). If correct, this would imply a biased process tending towards extremes, rather than gradual disarticulation. By contrast, skeletal completeness is more even, with 39% of specimens falling into class 0, ca 22% into class 1, and the remainder being almost equally split between the remaining classes. This pattern evokes a rather intuitive trend of progressive dismembering of the carcass, which in most cases nonetheless led to a notable reduction in completeness.

**Fig 7:**
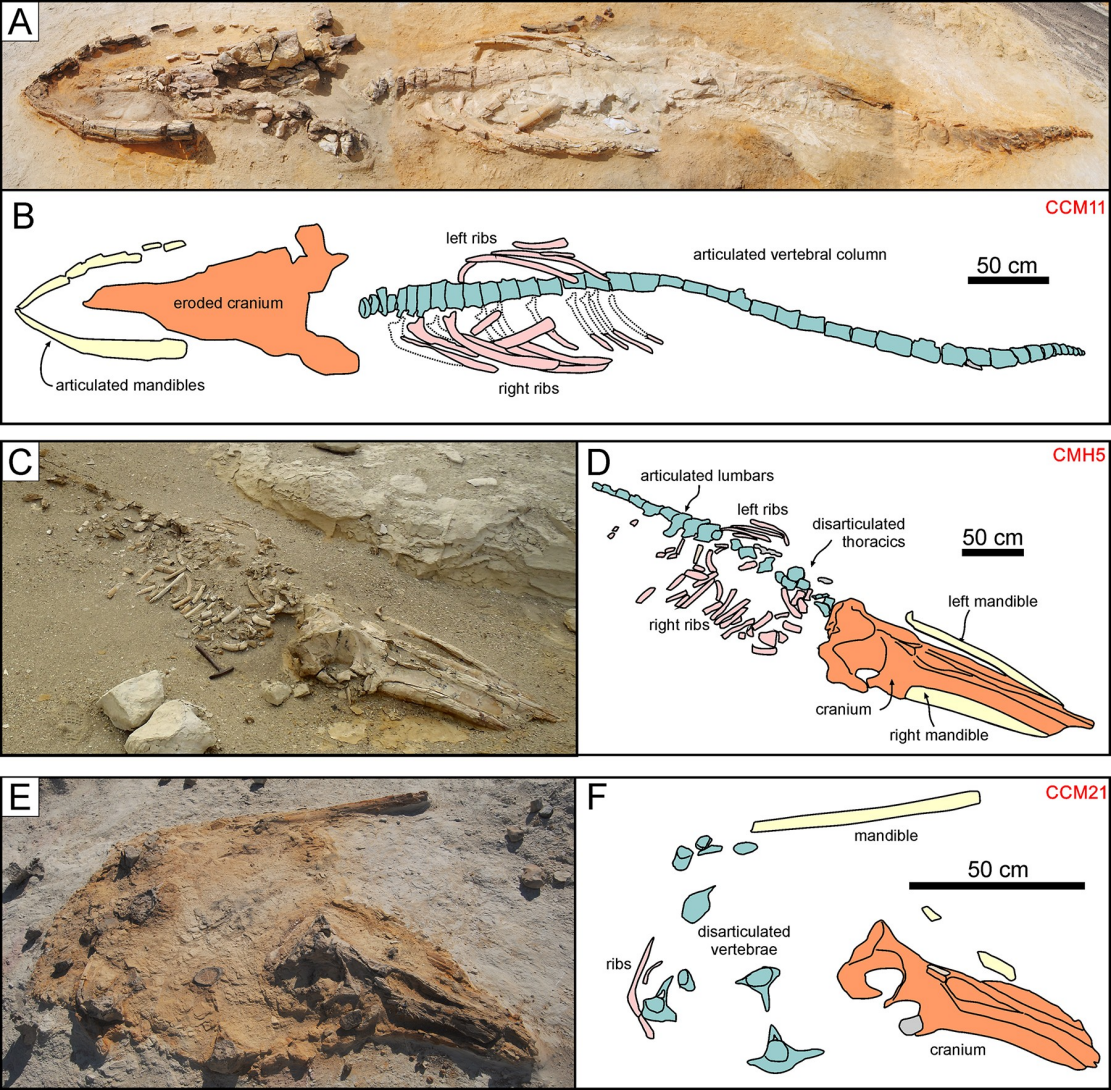
Cetotheriid mysticetes. Cetotheriid mysticete specimens from various localities and horizons of the Pisco Formation, displaying different degrees of articulation and completeness, and diverse preservational features. A, B) Field photograph (A) and explanatory line drawing (B) of CCM11, skeleton of Cetotheriidae indet. from P1 diatomaceous silts exposed at Cerro Colorado, preserved in ventral disposition, exhibiting high degrees of articulation and completeness (4,4). The dashed red circle indicates the occurrence of a dense aggregate of skeletal and dermal fish remains, here interpreted as comprising a fossilised gut content, posterior to the left posterior ribs of the whale. C,D) Field photograph (C) and explanatory line drawing (D) of CMH5, skeleton of Cetotheriidae indet. from P1 diatomaceous silts exposed at Cerros la Mama y la Hija, preserved in dorsal disposition, exhibiting a moderately high degree of articulation and a high degree of completeness (3,4). E, F) Field photograph (E) and explanatory line drawing (F) of CCM21, skeleton of Cetotheriidae indet. from P1 diatomites exposed at Cerro Colorado, exhibiting a very low degree of articulation and a moderately high degree of completeness (0,3). Panel E after Gariboldi et al. [23].

**Fig 8:**
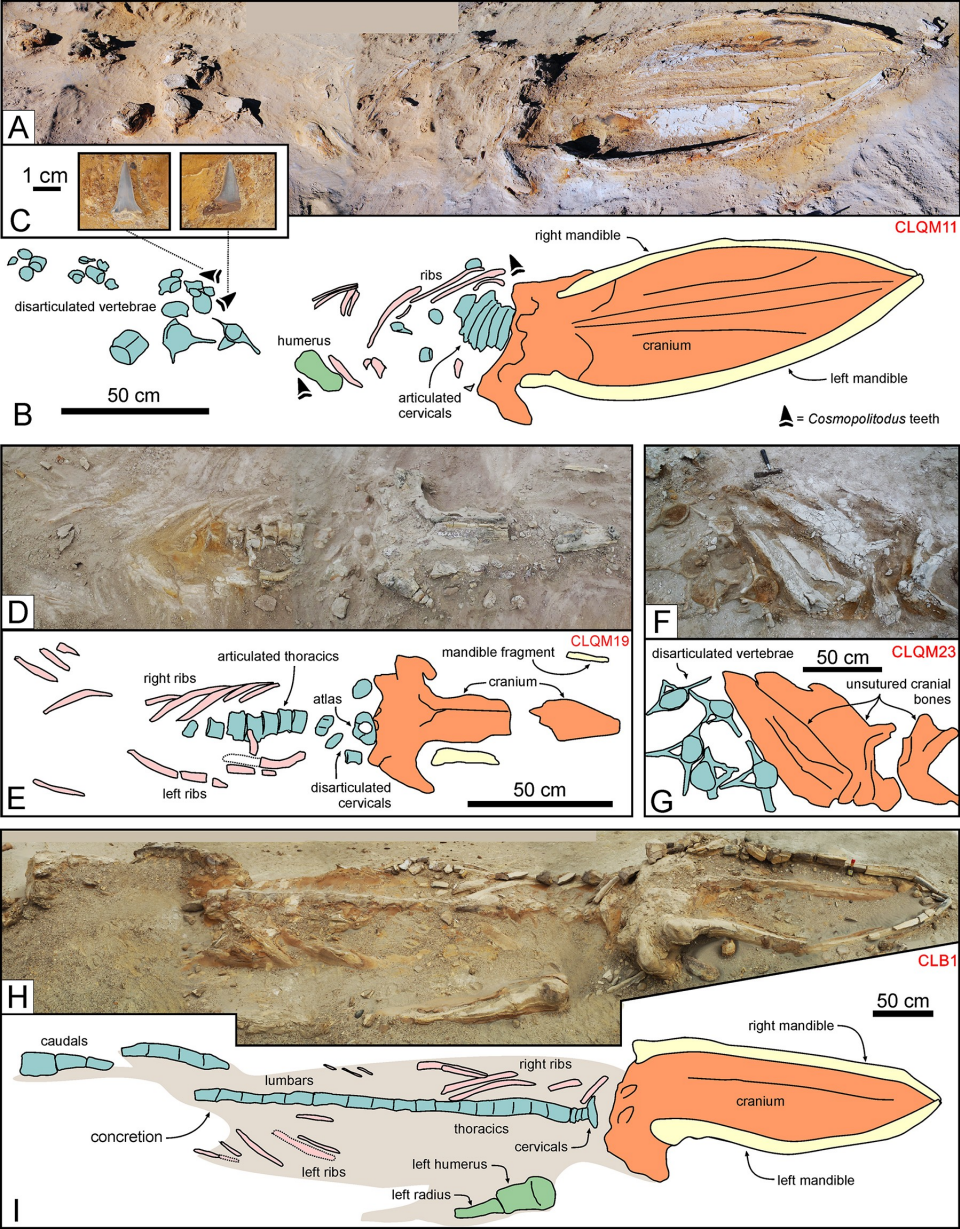
Balaenopteroid mysticetes. Balaenopteroid mysticete specimens from various localities and horizons of the Pisco Formation, displaying different degrees of articulation and completeness, and diverse preservational features. A, B) Field photograph (A) and explanatory line drawing (B) of CLQM11, skeleton of Balaenopteridae indet. from P2 diatomites exposed at Cerro los Quesos East, preserved in dorsal disposition, exhibiting a moderate degree of articulation and a moderately high degree of completeness (2,3). Note the presence of shark teeth preserved in close association with the whale bones (C). D, E) Field photograph (D) and explanatory line drawing (E) of CLQM19, skeleton of cf. *Piscobalaena* sp. from P2 diatomites exposed at Cerro los Quesos East, preserved in ventral disposition, exhibiting moderately high degrees of articulation and completeness (3,3). F, G) Field photograph (F) and explanatory line drawing (G) of CLQM23, skeleton of Mysticeti indet. from P2 diatomites exposed at Cerro los Quesos East (disposition not ascertained), exhibiting a very low degree of articulation and a moderate degree of completeness (0,2). Note the disposition of the lumbar vertebrae, having their neural spines and transverse processes parallel to the bedding plane, and the unfused cranial sutures. H, I) Field photograph (H) and explanatory line drawing (I) of CLB1, skeleton of Balaenopteridae indet. from P2 sands exposed at Cerro la Bruja, preserved in ventral disposition, exhibiting a moderately high degree of articulation and a high degree of completeness (3,4). The right baleen rack is preserved in this specimen, although not visible (i.e., hidden by the right mandible) in panel H.

**Fig 9:**
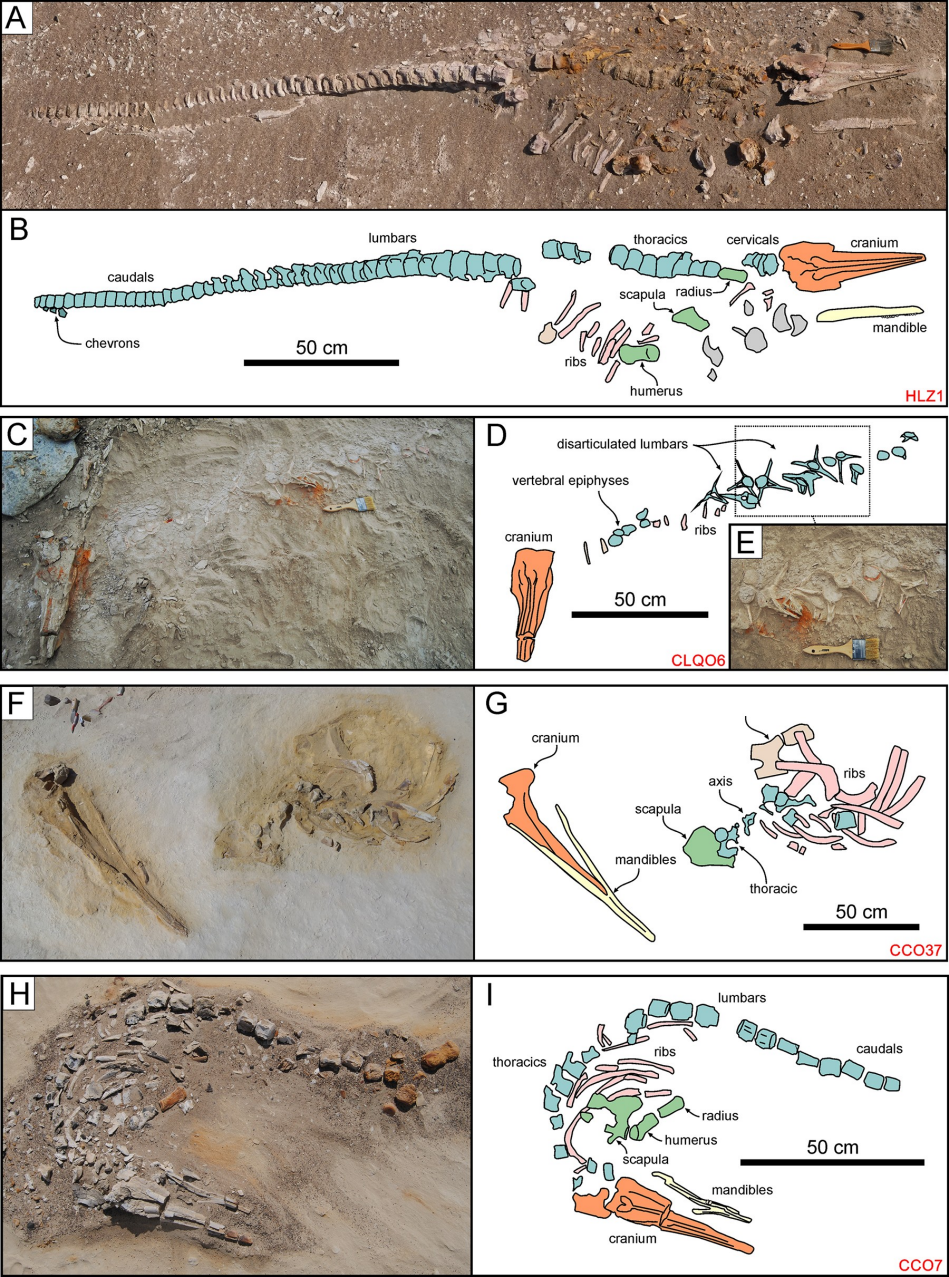
Odontocentes. Odontocete specimens from various localities and horizons of the Pisco Formation, displaying different degrees of articulation and completeness, and diverse preservational features. A, B) Field photograph (A) and explanatory line drawing (B) of HLZ1, skeleton of a Phocoenidae indet. from P2 diatomaceous silts exposed at Cerro Hueco la Zorra, preserved in lateral disposition, exhibiting high degrees of articulation and completeness (4,4). C, D, E) Field photographs (C, E) and explanatory line drawing (D) of CLQO6, skeleton of a Phocoenidae indet. from P2 diatomites exposed at Cerro Los Quesos East, preserved in dorsal disposition, exhibiting a very low degree of articulation and a moderately high degree of completeness (0,3). Note the disposition of the lumbar vertebrae, having their neural spines and transverse processes parallel to the bedding plane (E). F, G) Field photograph (F) and explanatory line drawing (G) of CCO37, skeleton of *Messapicetus gregarius* from P1 diatomites exposed at Cerro Colorado, preserved in lateral disposition, exhibiting moderate degrees of articulation and completeness (2,2). H, I) Field photograph (H) and explanatory line drawing (I) of CCO7, skeleton of cf. Kentriodontidae indet. from P1 sands exposed at Cerro Colorado, preserved in dorsal disposition, exhibiting high degrees of articulation and completeness (4,4). Panel H after Bianucci et al. [18].

**Fig 10:**
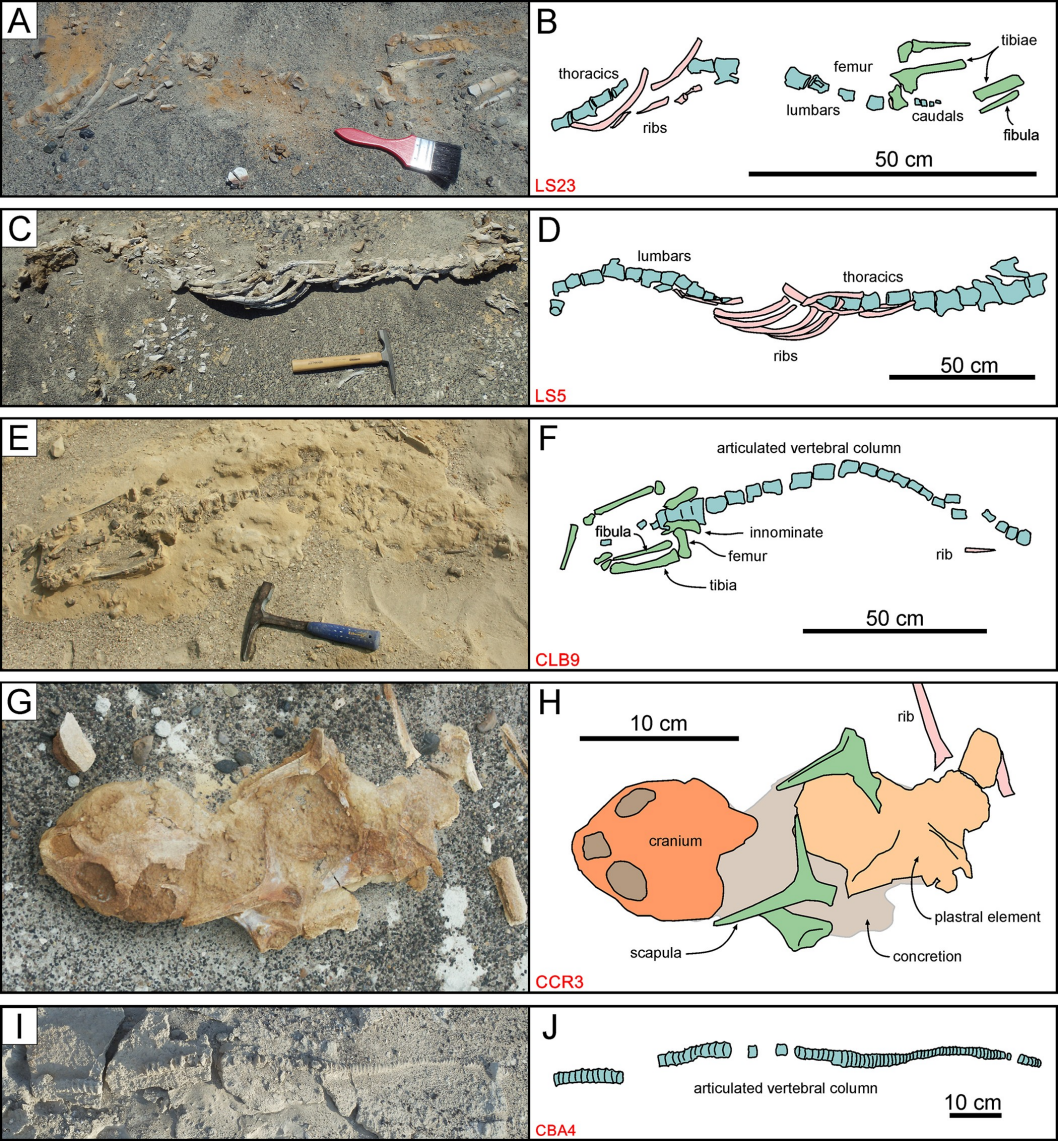
Non-cetacean vertebrates. Pinniped, reptile, and shark specimens from various localities and horizons of the Pisco Formation, displaying different degrees of articulation and completeness, and diverse preservational features. A, B) Field photograph (A) and explanatory line drawing (B) of LS23, skeleton of Phocidae indet. from P2 sands exposed at Laguna Seca (ex situ find). C,D) Field photograph (C) and explanatory line drawing (D) of LS5, skeleton of Phocidae indet. from P2 sands exposed at Laguna Seca, preserved in dorsal disposition, exhibiting a high degree of articulation and a moderately high degree of completeness (4,3). E, F) Field photograph (E) and explanatory line drawing (F) of CLB9, skeleton of Phocidae indet. from P2 sands exposed at Cerro la Bruja, preserved in dorsal disposition, exhibiting a moderately high degree of articulation and a moderate degree of completeness (3,2). G, H) Field photograph (G) and explanatory line drawing (H) of CCR3, skeleton of *Pacifichelys urbinai* from P1 diatomites exposed at Cerro Colorado, preserved in dorsal disposition, exhibiting a low degree of articulation and a moderate degree of completeness (1,2). Note the presence of an external concretion entombing the turtle remains. I, J) Field photograph (I) and explanatory line drawing (J) of CBA4, skeleton of Carcharhinidae indet. from P2 diatomaceous silts exposed at Cerro Ballena (disposition not ascertained), exhibiting a high degree of articulation and a low degree of completeness (4,1).

Mysticetes show the highest degree of articulation (Fig 6B), with >40% being completely or moderately articulated (Figs 7 and 8). The reverse is true of odontocetes, with >60% of them being completely disarticulated (Fig 9). Pinnipeds follow the odontocete pattern, but more of them (29% vs 23%) are preserved fully articulated (Fig 10A–10F). Across all groups, articulation class 0 accounts for >50%, whereas classes 1–3 together account for <20%. This pattern further supports the notion that different biostratinomic paths and processes lead to the preservation of either remarkably articulated or fully disarticulated specimens.

Variations in skeletal completeness are comparable across all taxonomic groups (Fig 6B), with intermediate completeness classes (1–3) accounting for a relatively large number of specimens. Mysticetes stand out for being comparatively well preserved, with classes 4 and 3 accounting for almost 40% of the specimens, and >20% being fully complete. Odontocetes and pinnipeds again show similar proportions, with classes 4 and 3 together accounting for <30%; however, the percentage of fully complete pinnipeds is half that of odontocetes. The high percentage of mysticetes (33%) and odontocetes (37%) falling into class 0 at least partly reflects isolated crania, which probably became detached from floating carcasses. By contrast, class 0 in pinnipeds (also 37%) is dominated by isolated postcranial bones. Overall, mysticetes tend to be more complete than odontocetes, which in turn tend to be more complete than pinnipeds. This pattern likely reflects different thresholds or timings of dismemberment, perhaps as a function of total body size, relative skull size (Fig 3), integument thickness, and/or tendency to float or re-float after sinking. Differences in floating propensity across extant cetaceans [63, 64] do not easily conform to our observations, which also include extinct taxa (e.g. cetotheriids [19, 26, 28]) for which the tendency to float is unknown.

Overall, diatomaceous silts yield specimens with the highest degree of articulation (Fig 6C), with class 4 accounting for 40% of the total number of specimens, and classes 4 and 3 together reaching nearly 60%. By contrast, silts preserve the least articulated specimens, with class 0 accounting for about 70%; however, the total number of specimens (26) recovered from silts is low, which casts some doubt on the robustness of this result. Skeletal completeness also peaks in the diatomaceous silts, with 19% of all specimens being fully complete; silts again perform worst, with fully complete specimens only accounting for about 4%. P1 contains the highest number of articulated specimens, with classes 4 and 3 accounting for >40%, whereas they represent <30% in P0 (Fig 6D). Conversely, P1 displays the lowest number of fully disarticulated specimens (48%), compared with 64% in P0.

Overall, articulation and completeness follow similar patterns (bimodal vs progressive) across taxa and lithologies, but are at times decoupled in terms of the relative importance. In general, distal diatom-rich deposits (diatomites and diatomaceous silts) provide better conditions for preservation, perhaps because their high sedimentation rates and soft texture prevent carcasses from re-floating [14]. Surprisingly, sands appear to be more favourable for preservation than silts, possibly as a result of stranded animals accumulating ashore [63]; however, conclusions are again limited by the small number of specimens known from silts.

**Fig 11:**
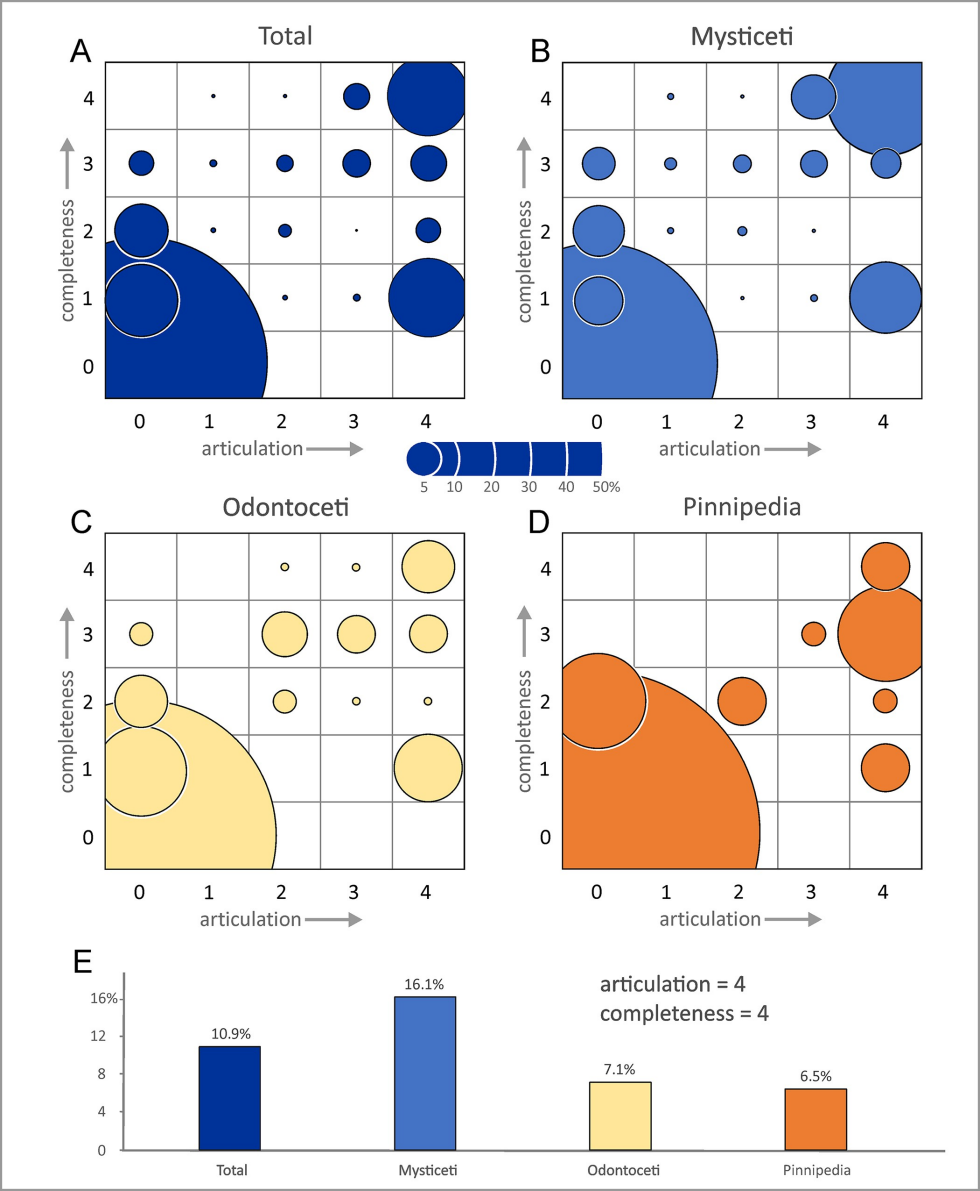
Articulation and completeness relative to taxonomy. A, B, C, D) Bubble plots of articulation versus completeness for the fossil marine vertebrates from the Pisco Formation with respect to the total vertebrate assemblage and the main taxonomic groups of marine mammals. The bar diagram (E) illustrates the percentages of specimens characterised by exquisite degrees of articulation and completeness (4,4).

Articulation and completeness are decoupled across the three allomembers. Thus, completeness in P0 is relatively high whereas articulation is low, perhaps again reflecting shore-based concentration of stranded skeletons. Similarly, both measures are largely decoupled across mysticetes, odontocetes and pinnipeds. Extreme values (classes 0 and 4) correlate well, with the large percentage of highly articulated and complete mysticetes (16.1%, more than twice that of odontocetes and pinnipeds) once again suggesting an intrinsic taphonomic bias related to size, integument thickness, and/or tendency to float or re-float after sinking (Fig 11).

By contrast, intermediate states form two distinct clusters. The first includes specimens with a high degree of articulation but variable completeness, suggesting progressive dismemberment of floating carcasses via detachment of the cranium, mandibles, and limbs. Some specimens do also show a modest loss of articulation, which might be explained by disarticulated bones remaining trapped inside the decaying integument [14, 63]. The second cluster comprises specimens with a low degree of articulation and variable (but mostly low or very low) completeness. We suggest that this cluster reflects two separate processes: partial skeletons with a relatively high completeness score (2–3) might derive from specimens that remained exposed on the seafloor after deposition, whereas lower values (0–1) might represent debris (mostly isolated skulls, some of them with mandibles) left by floating carcasses.

**Fig 12:**
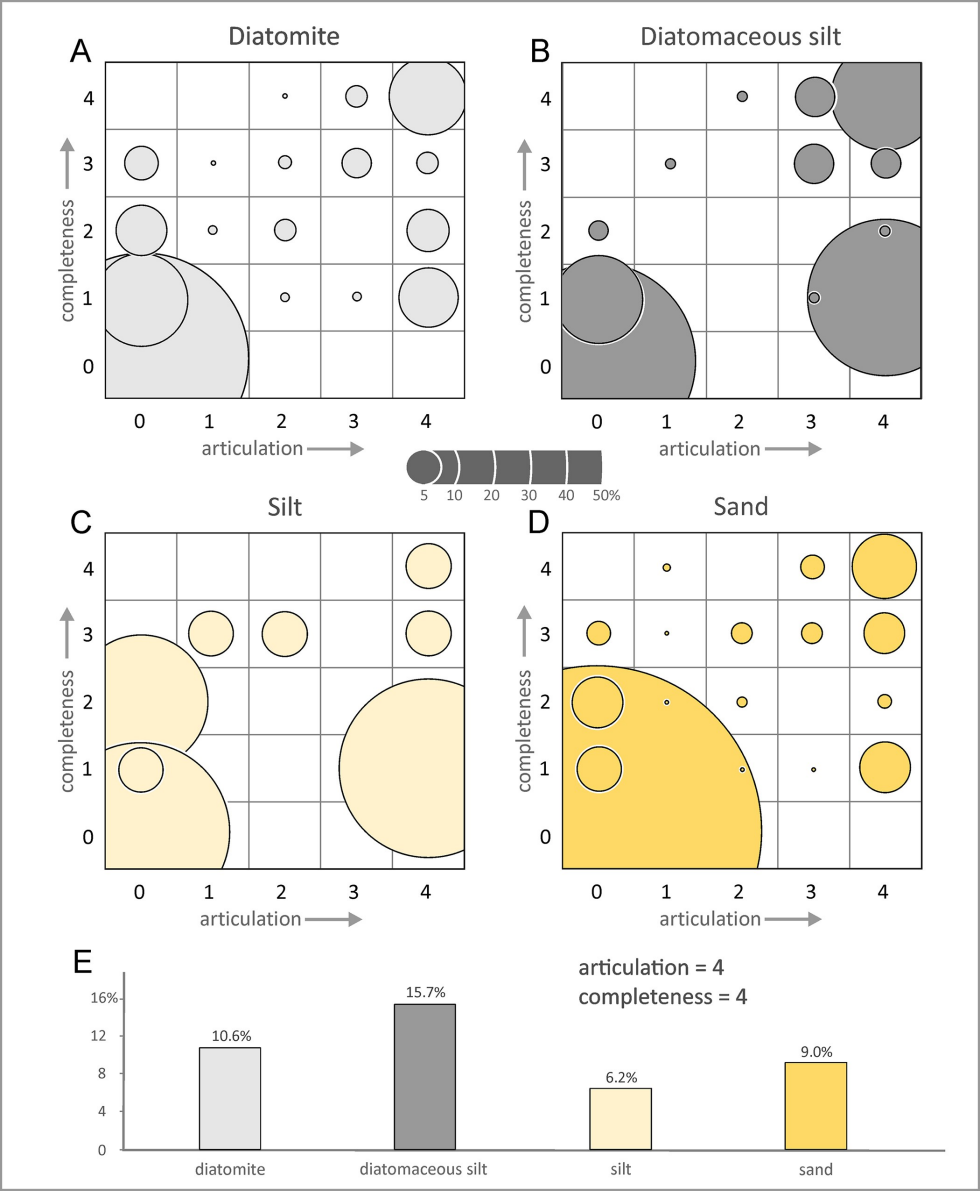
Articulation and completeness relative to lithology. A, B, C, D) Bubble plots of articulation versus completeness for the fossil marine vertebrates from the Pisco Formation with respect to the four different entombing lithologies. The bar diagram (E) illustrates the percentages of specimens characterised by exquisite degrees of articulation and completeness (4,4).

Extreme degrees of articulation and completeness (classes 0 and 4) also correlate across different lithologies, with diatomaceous silts–but not silts–standing out for their large percentage of specimens with moderate–high values (Fig 12). This difference may reflect the soupy texture of diatomaceous silts, which may facilitate carcasses sinking into and becoming trapped in the sediment with comparatively little chance of re-floating.

### Disposition

**Fig 13:**
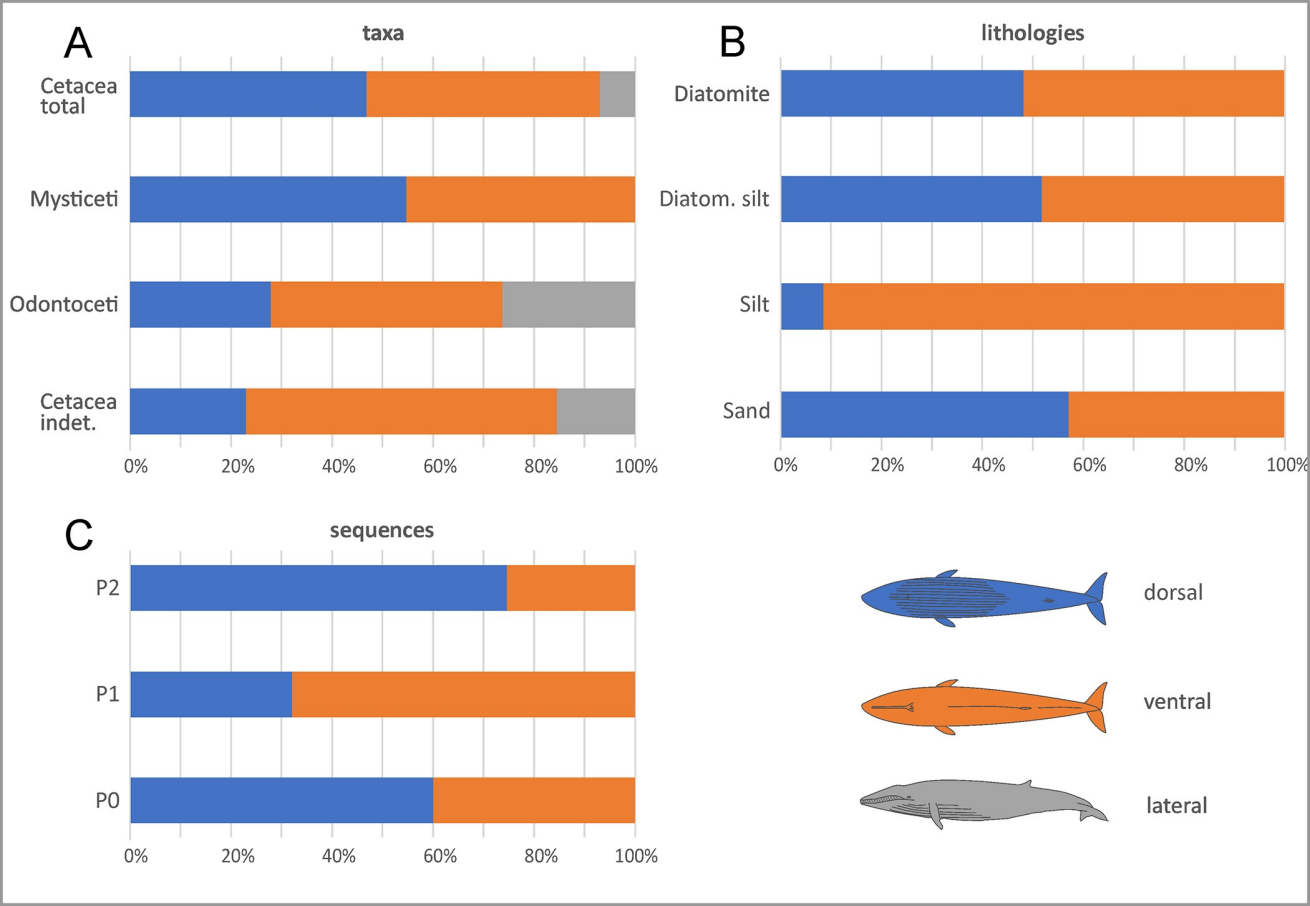
Patterns of disposition. Bar diagrams showing the disposition (dorsal, ventral or lateral) distribution of the fossil marine vertebrates from the Pisco Formation with respect to various samples: the main taxonomic groups of cetaceans (A), the different entombing lithologies (B), and the different host sequences (C).

Numerous mysticetes and odontocetes are preserved belly-up (Fig 13A), suggesting that they floated before sinking to the seafloor. During decay, gases accumulate in the abdominal cavity of floating carcasses (especially whales), causing the body to bloat and eventually flip [14, 64]. Floating appears common across all lithologies and sequences except P2, which mostly records an offshore environment with a soupy diatomaceous substrate that might have trapped specimens on the seafloor (Fig 13B and 13C). Mysticetes are never preserved lateral side up, perhaps because their large size prevented rolling, or because their relatively flat skulls naturally assumed a horizontal position as the carcass disintegrated.

### Orientation

**Fig 14:**
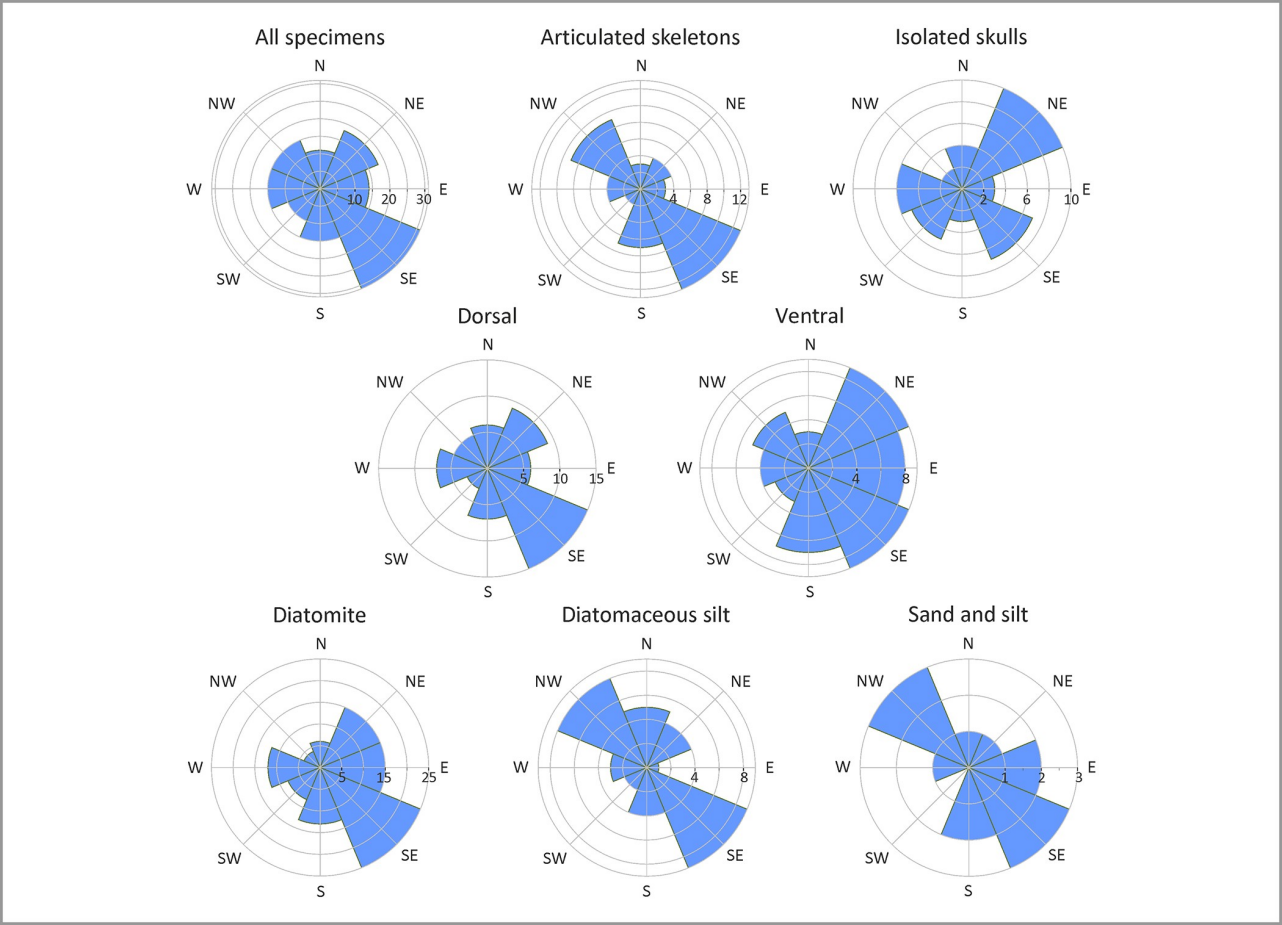
Patterns of orientation. Rose diagrams showing the orientation of the fossil marine mammals from the Pisco Formation, considering the total assemblage at Cerro Colorado and Cerro Los Quesos East, the articulated skeletons, the isolated skulls, the specimens preserved in dorsal and ventral disposition, and the specimens preserved in the different lithologies. Note the remarkable southeastward orientation of all the specimens, especially the fully articulated ones and those preserved in dorsal disposition. The isolated skulls are preferentially oriented northeastward. In the diatomites, the specimens are mostly oriented southeastward, whereas the specimens that are preserved in the sands, silts and diatomaceous silts display a preferential, NW-SE bimodal orientation.

Most skeletons are oriented NW-SE (Fig 14), roughly parallel to both the modern and the Miocene coast (Fig 1B). This is especially true of articulated and/or dorsal-up specimens, with the latter being preferentially oriented south-eastward. We suggest that this pattern may reflect the direction of the north-westward Peruvian Coastal Current, which flows parallel to the coast at a depth of <80 m and could plausibly have reoriented the floating or sinking carcasses using the head as a fulcrum [65]. Given their orientation, predominantly dorsal disposition and high degree of articulation, skeletons with a SE orientation probably did not re-float following deposition on the sea floor. Specimens preserved ventral-side up and isolated skulls show a more complicated orientation pattern and, thus, transport history.

Specimens across all lithologies are mostly parallel to the coast (i.e. NW-SE; Fig 14), but they tend to be unimodally oriented south-eastward in the diatomites. This difference may be related to water depth, with carcasses sinking in deeper settings (i.e. those producing diatomites) having more time to become re-oriented by the Peruvian Coastal Current, and the nature of the diatomaceous sediments themselves, which prevents the re-oriented carcasses from re-floating.

### Shark bite marks

Despite a great abundance of shark teeth, we encountered bite marks–dentalites *sensu* Hunt et al. [66]–on just 13 (1.6%) of the 821 marine mammals we examined. A previous study by Esperante et al. [14] reported an even lower frequency, with just three whale fossils (a skull, vertebra, and rib, respectively) out of the whole surveyed assemblage showing bite marks, whereas all the 571 cetacean specimens they examined in detail did not. In both cases, the observed numbers are likely an underestimate, as bite marks could have remained hidden by sediment. Even so, bite marks in the Pisco strata overall appear rarer than in the underlying Chilcatay Formation [56].

Specimens with bite marks tend to be relatively complete and articulated, and include 7 mysticetes (4 cetotheriids, 3 indeterminate), 3 odontocetes (1 phocoenid, 2 indeterminate delphinidans), and 3 indeterminate cetaceans. Surprisingly, there are no bite marks on any of the pinniped specimens, even though seals are commonly targeted by modern mammal-eating sharks [67]. The frequency of bite marks varies across allomembers, reaching 3/126 specimens (2.4%) in P0, 2/324 (0.6%) in P1, and 3/342 (0.9%) in P2. One notable outlier consists of likely P2 strata exposed at Laguna Seca, where bite marks appear frequent (5/41; 12.2%).

Specimens from P0 include 2 mysticetes and 1 odontocete from the localities of Dos Cerritos and Cerro Submarino. The most significant (MLP1) consists of a rather eroded partial delphinidan skeleton bearing several incisions on the ribs (Collareta et al. [35]: Fig 4C). Most of these traces are short (<1 cm) and represent morphological-genetic types I or II (*sensu* Cigala Fulgosi [68] and Bianucci et al. [69]). More prominent is a cluster of three type III incisions with a maximum length of ca 2 cm and several parallel grooves implying a tooth with serrated edges. Although mega-toothed sharks such as *Carcharocles megalodon* cannot be ruled out, the size and depth of the bite marks are more suggestive of large carcharhinids like *Carcharhinus* cf. *leucas* or *Galeocerdo aduncus*, both of which occur in P0 [35, 70].

**Fig 15:**
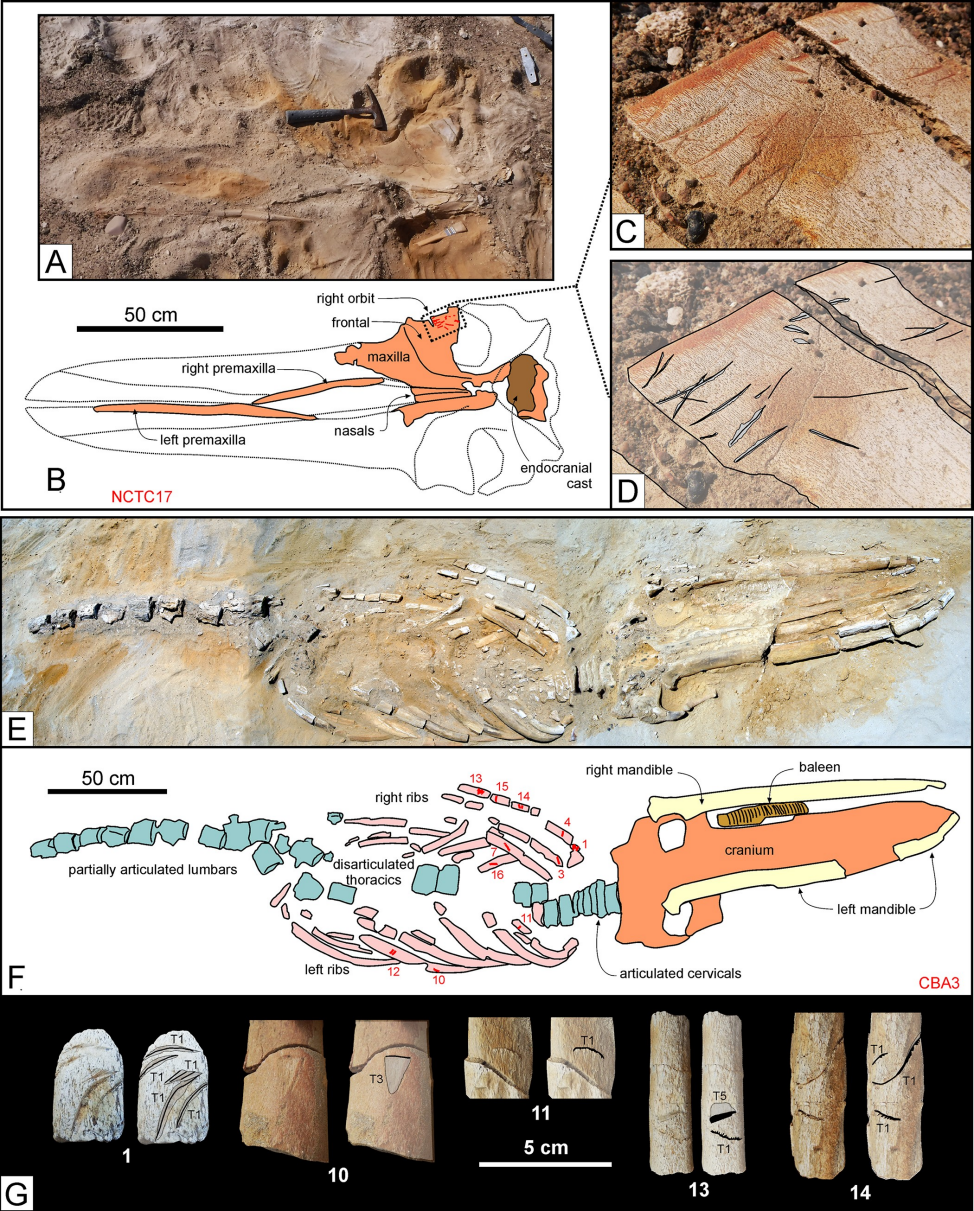
Shark bite marks. Shark bite marks on fossil cetacean specimens from various localities and horizons of the Pisco Formation. A, B, C, D) Field photographs (A, C) and explanatory line drawings (B, D) of the cranium and mandibles of NCTC17, skeleton of Cetotheriidae indet. from P1 diatomites exposed at Corre Viento, preserved in dorsal disposition. Note the presence of several unserrated, shallowly impressed, type I or type II incisions on the dorsal surface of the supraorbital process of the right frontal. E, F, G) Field photographs (E, G) and explanatory line drawings (F, G) of CBA3, skeleton of *Piscobalaena nana* from P2 diatomaceous silts exposed at Cerro Ballena [26], preserved in ventral disposition. Note the abundance of shark bite marks on the whale’s rib cage (F), and especially on the anteriormost right ribs (G). The numbers in panel G refer to the labels in panel F. T1, T2, T3, and T5 in panel G refer to the different shark bite mark types as defined in the main text.

Of the two specimens from P1, one (CCO5) comes from Cerro Colorado and consists of a weathered but almost complete skeleton of a kentriodontid-like odontocete with straight or slightly sinuous type I or type II bite marks on the ribs. The other (NCTC17) comes from the uppermost portion of P1 exposed at Corre Viento, and consists of a well-preserved juvenile belonging to an undescribed species of cetotheriid [28]. The skull of this specimen bears >10 type I or type II incisions on the dorsal surface of the right supraorbital process of the frontal (Fig 15A–15D). Most of these tooth marks are shallow, relatively straight, and up to 3.5 cm long. In addition, there are some deeper, shorter punctures, probably made by the tip of the impacting tooth. Together with the lack of serrations, the length and shape of the incisions point to a large lamniform like *Cosmopolitodus hastalis* or *C*. *plicatilis*, both of which occur in P1 [20].

Finally, the most impressive specimen from P2 (CBA3) comes from Cerro Ballena and consists of an almost complete skeleton of the small cetotheriid mysticete *Piscobalaena nana*. The latter lies ventral-side up and is both articulated and largely complete, lacking only the forelimbs, hyoid, sternum, and the posteriormost vertebrae (Fig 15E). In addition, this specimen stands out for preserving a phosphatised baleen rack [26] (see also Baleen preservation). Bite marks occur on both sides of the thorax (Fig 15F and 15G), with the first right rib showing deep, curved, closely spaced, up to 3 cm long type II gouges, as well as shorter type I incisions and pervasive type V damage (*sensu* Collareta et al. [29]). Their producer was large and armed with labiolingually flattened, non-serrated teeth, as seen in *Cosmopolitodus hastalis* or *C*. *plicatilis*. At Cerro Los Quesos, comparably aged deposits (member F; [71]) have yielded a large balaenopterid-like specimen (CLQM51) with deep bite marks on one rib that also clearly match *Cosmopolitodus*.

### Associated shark teeth

Associations of shark teeth and cetacean skeletons are relatively common in the Pisco Formation [14, 17] and represent indirect evidence of trophic interactions, albeit of a weaker kind than bite marks [69]. Obtaining quantitative data on their abundance is challenging, however, given (i) the need for long and painstaking excavations, especially in the case of large whales; and (ii) the fact that illegal collecting has notably reduced the number of large shark teeth over the course of our field campaigns [18]. To ameliorate these problems, we focused our efforts on just one promising locality per allomember.

At Cerro Los Quesos East, we excavated 31 mysticete skeletons from the P2, 8 of which (26%, including the aforementioned CLQM51) were associated with shark teeth. In most cases, the latter comprise just one or two teeth belonging to *Carcharhinus*, *Carcharocles* or *Cosmopolitodus*. Considering that baleen whale skeletons often cover large areas, as well as the occurrence of isolated shark teeth elsewhere in the P2 diatomites, the trophic significance of such limited associations is doubtful. A better case can be made for assemblages involving several shark specimens. Thus, for example, we found a balaenopterid (CLQM11) with six teeth–including some belonging to *Cosmopolitodus hastalis*–preserved close to its skull and vertebrae (Fig 8A–8C).

Another large, partially articulated balaenopterid (CLQM7) was associated with as many as 39 teeth, found mostly around the skull and mandibles. About half of the assemblage consists of bull sharks (*Carcharhinus leucas*) and eagle rays (Myliobatidae indet.), with the remaining teeth belonging to *Cosmopolitodus hastalis*, one or more species of requiem sharks (*Carcharhinus* spp.), and the extinct tiger shark *Physogaleus contortus*. We presume that none of these species actively preyed on the much larger whale, but they plausibly contributed to scavenging.

Modern observations show that a single floating cetacean carcass may support a range of scavengers, including carcharhinids like bull and tiger sharks (*Galeocerdo cuvier*), and lamnids like shortfin makos (*Isurus oxyrinchus*) and great whites (*Carcharodon carcharias*), the latter being the closest living relatives of *C*. *hastalis* [72–75]. Tiger and bull sharks frequently target the blubber-rich throat region of extant mysticetes [75, 76], with teeth occasionally getting stuck in the bitten items [73]. Similar behaviours may account for the high concentration of teeth associated with CLQM7, but fail to explain the absence of bite marks on the whale bones. Likewise, the presence of myliobatids, largely durophagous rays not known to forage on marine mammal carrion, remains difficult to interpret.

Surveys at Cerro Submarino (P0) and Cerro Colorado (P1) revealed only one cetacean associated with shark teeth at either locality, despite the presence of some notable shark tooth-bearing beds [20]. This observation is consistent with the most productive of these beds reflecting a communal elasmobranch nursery dominated by mesopredators [21], which presumably did not regularly target marine mammals.

### Associated macro-invertebrates

Associations of invertebrates with vertebrate remains may occur in both shallow [77–81] and deep [82] marine settings, and have been studied both in the present [83–87] and in the fossil record [88, 89]. Invertebrates are particularly common on and around whale carcasses, where they may account for distinct ‘whale-fall’ communities [90, 91].

Evidence for the presence of whale-fall communities in the Pisco Formation is scarce [14]. This is confirmed by our own observations, which show that <10% of vertebrate fossils is found together with an invertebrate assemblage. Where present, the latter is usually moderately diverse, being comprised of mollusc and/or barnacles, and only rarely can be clearly tied to the presence of the carcass on the seafloor. In addition, fossil vertebrates are occasionally found in shell beds that do not represent whale-fall communities as such.

**Fig 16:**
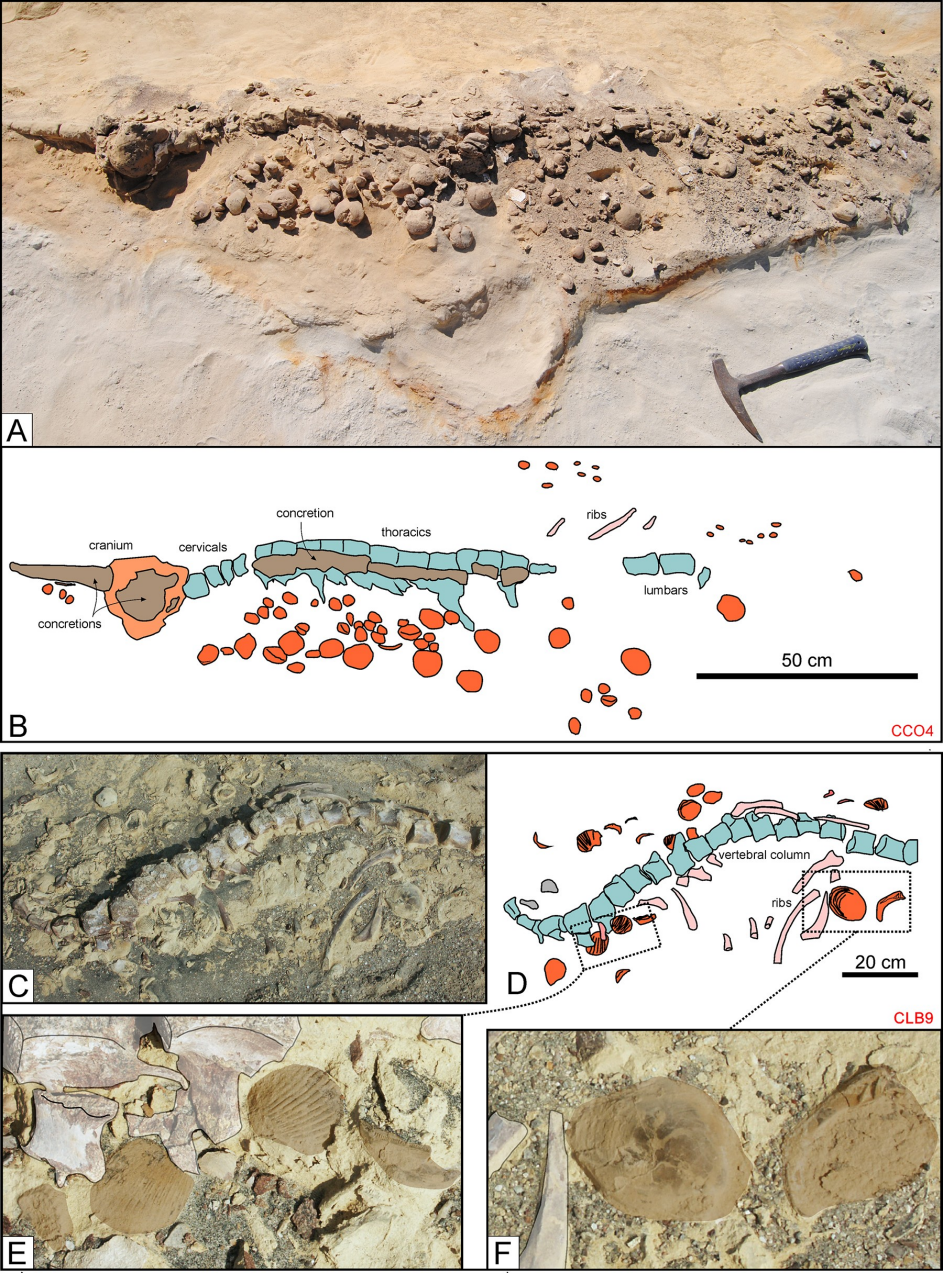
Associated macro-invertebrates (I). Invertebrates associated with fossil cetacean specimens from various localities and horizons of the Pisco Formation. A, B) Field photograph (A) and explanatory line drawing (B) of CCO4, skeleton of cf. Kentriodontidae indet. from P1 diatomaceous silts exposed at Cerro Colorado, preserved in lateral disposition. Note the internal moulds of the bivalves *Dosinia ponderosa* and *Anadara sechurana* that surround the skeleton. C, D, E, F) Field photographs (C, E, F) and corresponding line drawing (D) of CLB9, skeleton of Phocidae indet. from P2 sands exposed at Cerro la Bruja, lying on a shell bed. Note the close-ups of bivalve shells referred to Arcidae (E) and Ostreidae and Veneridae (F).

**Fig 17:**
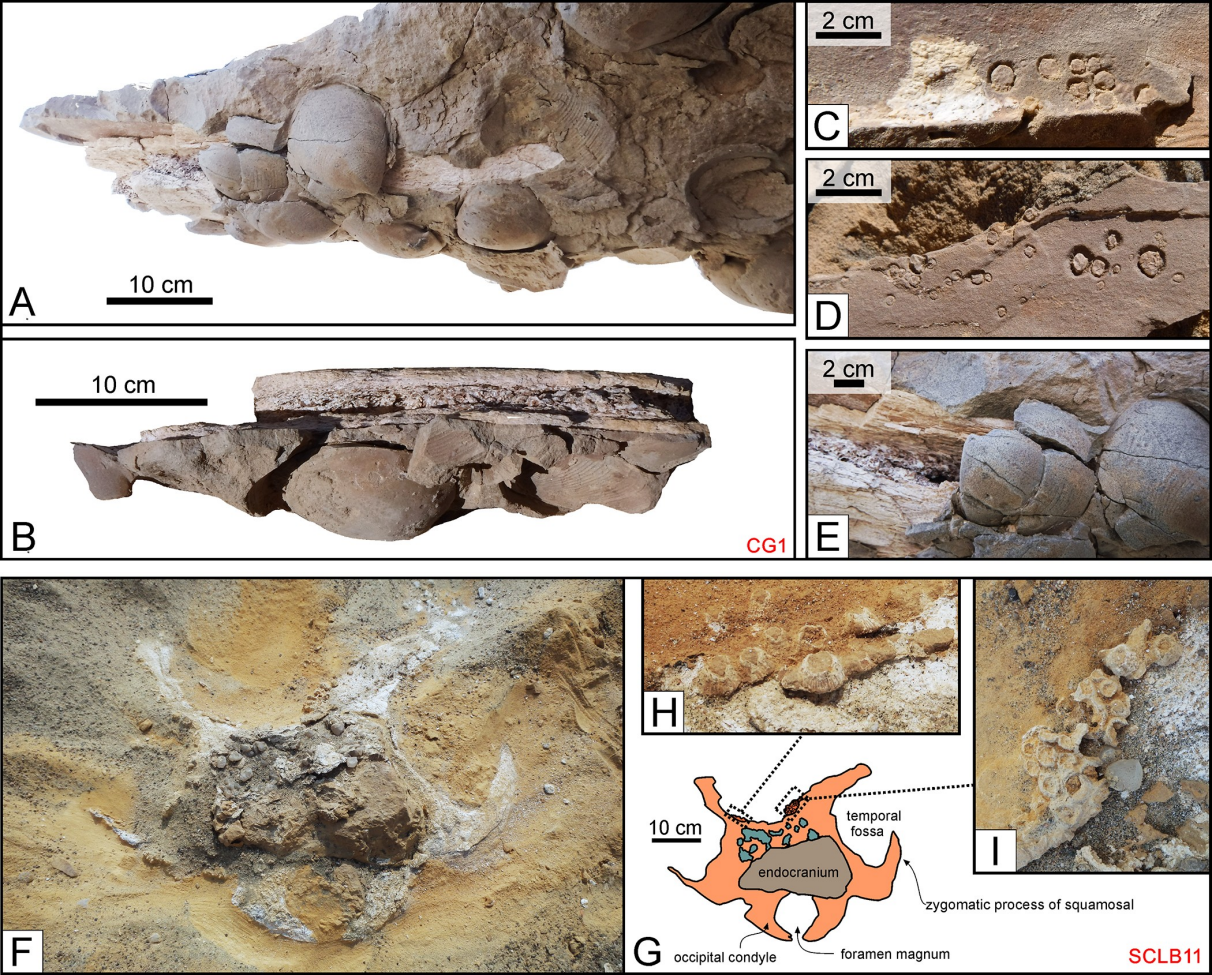
Associated macro-invertebrates (II). Invertebrates associated with fossil cetacean specimens from various localities and horizons of the Pisco Formation. A, B, C, D, E) Field photographs of CG1, skeleton of Cetotheriidae indet. from P1 sands exposed at Cerro Geoduck, preserved in ventral disposition. CG1 lays on a monospecific shell bed of *Dosinia ponderosa* (A, B, E). (C, D) Close-up of dolomite moulds of balanid barnacles encrusting the elongated bones of the cetacean. F, G, H, I) Field photographs (F, H, I) and corresponding line drawing (G) of SCLB11, skeleton of cf. Physeteroidea indet. from P1 sands exposed at Cerro la Bruja South, preserved in dorsal disposition. SCLB11 exhibits clusters of balanid barnacle shells along the posterior border of the supracranial basin and on the supraoccipital shield (H, I), as well as along the upper border of the left temporal fossa.

Examples for such chance associations come from the basal sandstones of both P1 and P2, where molluscs are preserved only as internal or external moulds [19, 70]. At Cerro Colorado (P1), an articulated and almost complete kentriodontid-like odontocete (CCO4) occurs in a shell bed dominated by *Dosinia ponderosa* and *Anadara sechurana* (Fig 16A and 16B). At the same locality, about two meters lower in the sequence, bivalve moulds representing the same genera occur near the cranium of a beaked whale (*Messapicetus*) skeleton (CCO43) [30]. The moulds are not in life position, again suggesting that the whale carcass came to rest on a shell bed. At Quebrada del Toro Chico (P2), a partially articulated mysticete is surrounded by numerous dolomite moulds of *Dosinia* and *Turritella*. At Cerro la Bruja (P2), a pinniped specimen (CLB9) is lying on a shell bed comprising arcids, ostreids, and venerids (Fig 16C–16F). Finally, at Cerro Geoduck (P1), southeast of Cerro Colorado, a poorly articulated mysticete skeleton (CG1) occurs inside a monospecific shell bed of *D*. *ponderosa* (Fig 17A, 17B and 17E).

Acorn barnacles tend to encrust clean bones in shallow marine environments [92, 93] and their aggregates and traces can be also found in the fossil record [4, 94, 95]. At Cerro Geoduck, the dolomite shell bed containing specimen CG1 also reveals moulds of balanid barnacles once attached to several of the bones (Fig 17C and 17D). At Cerro la Bruja South (P1), a sperm whale cranium (SCLB11) preserves clusters of balanids within the supracranial basin, along its posterior border, on the supraoccipital shield, and along the upper rim of the left temporal fossa (Fig 17F–17I). The presence of barnacles suggests the presence of bare bone, and thus decomposition of the overlying soft tissues, prior to burial. In addition, they imply a dynamic and well-oxygenated palaeoenvironment with high nutrient availability [96], as also indicated by obvious pre-burial erosion of the skull.

**Fig 18:**
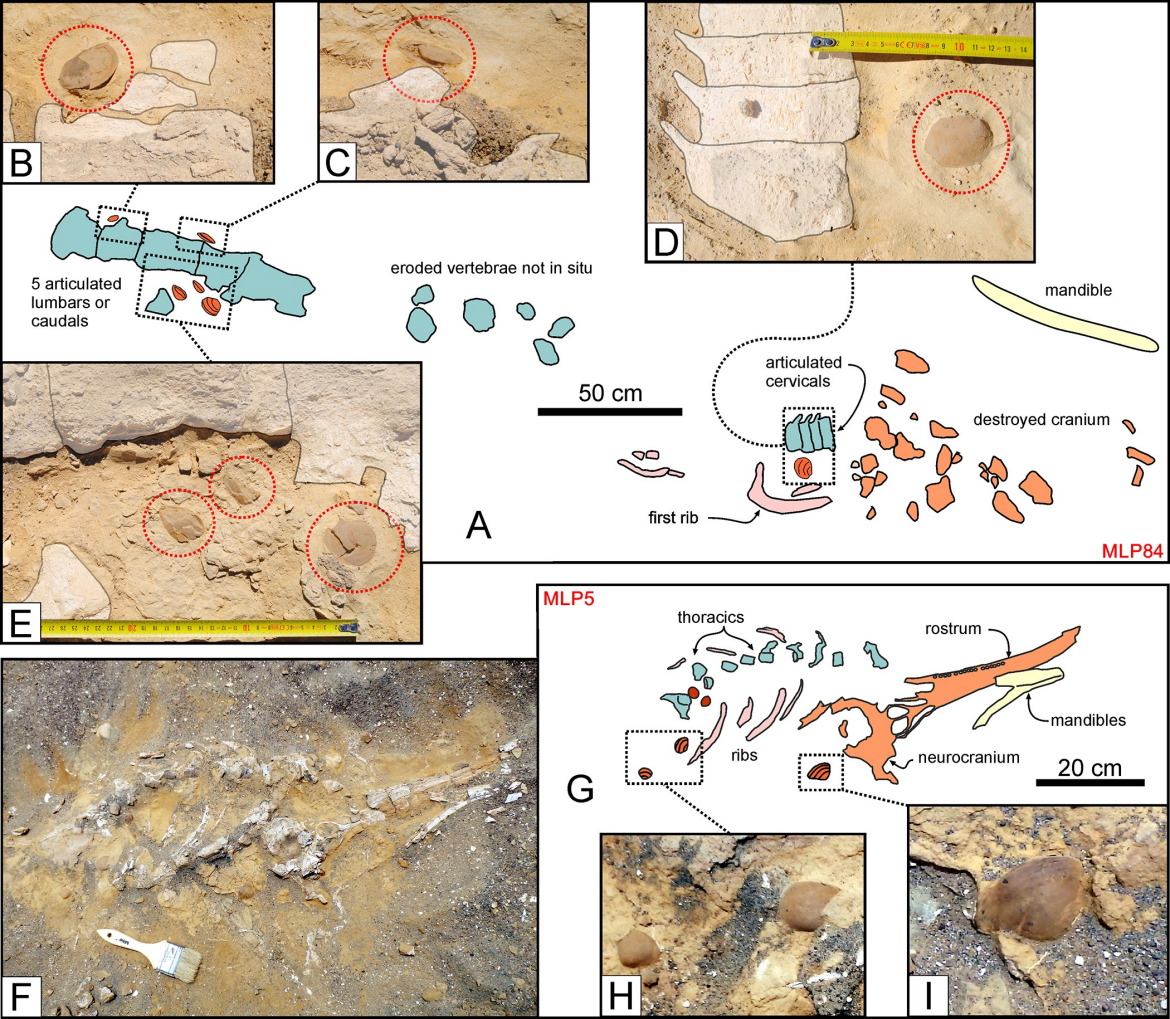
Associated macro-invertebrates (III). Invertebrates associated with fossil cetacean specimens from various localities and horizons of the Pisco Formation. A, B, C, D, E) Field photographs (B, C, D, E) and corresponding line drawing (A) of MLP84, skeleton of Mysticeti indet. from P0 sands exposed at Cerro Submarino, exhibiting several bivalve shells in the nearby sediment. The close-ups of *Miltha* specimens preserved near the lumbar and caudal vertebrae (B, C, E) and between the cervicals and the first rib (D) demonstrates that three of these bivalves are preserved in life position. F, G) Field photograph (F) and corresponding line drawing (G) of MLP5, skeleton of Kentriodontidae indet. from P0 sands exposed at Dos Cerritos, preserved in ventral disposition. H, I) Close-ups of three? *Dosinia* specimens that are associated to MLP5, including one shell (I) that is preserved in life position.

Genuine whale-fall communities are dominated by molluscs (especially bivalves) found in life position on or near the bones. Invertebrates are best preserved and most associated with cetaceans (9 cases) in the shoreface sandstones at the base of P0. At Cerro Submarino, several bivalves and a few gastropods occur near an almost fully disarticulated mysticete (MLP84), but not anywhere else within the same layer. Five bivalves of the genus *Miltha*, including three in life position, are located along the vertebral column (Fig 18A–18E), and suggest exposure of the bones on the seafloor. At the same locality, the vertebral columns of two (partially) articulated mysticetes (MLP75 and MLP86) are associated with small arcid bivalves and some gastropods, respectively. In addition, three bivalves resembling *Dosinia*, including one in life position, were each found with a partially articulated delphinidan (MLP5; Fig 18F–18I) and a fragmentary indeterminate cetacean (MLP20). Finally, another indeterminate cetacean (MLP89) preserves three gastropods, four *Miltha* sp. and one pectinid near or on its vertebrae and ribs, which together suggest a relatively long time of exposure on the seafloor.

Invertebrates are less common in the diatomaceous portions of P1 and P2, and were found on just four vertebrates. In the diatomaceous siltstones at Cerro Los Quesos East, a fragmentary phocoenid (CLQO7) is associated with six badly preserved casts of the semi-infaunal, suspension feeding bivalve *Hybolophus*. Similarly, specimens of the same genus, still in life position and in the same state of preservation, were found along the disarticulated vertebrae of a young indeterminate cetacean (CLQC46) (Gioncada et al. [40]: Fig 2). The presence of these bivalves, preserved as either gypsum/anhydrite casts or gypsum and Mn-Fe-enriched dolomite moulds, demonstrates that a whale-fall community settled in the sediment near the carcass.

Notwithstanding the presence of some molluscs-bones associations, there seem to be no typical whale-fall communities in the Pisco Formation. Fossil bones generally lack both macroinvertebrate marks like borings and signs of scavenging, except for some shark-induced dentalites (see Shark bite marks and Associated shark teeth). There is no evidence of the ‘zombie worm’ *Osedax* or other polychaetes, crustaceans other than barnacles, and sulphophilic bivalve taxa like *Adipicola*, *Calyptogena*, *Conchocele*, *Idas*, *Solemya*, and *Vesicomya* [82, 97–99]. On the contrary, both balanid barnacles and the arcid, crassatellid, lucinid, pectinid and venerid bivalves actually found in the Pisco Formation suggest that whales came to rest amid a ’normal’ benthos [100]. In the context of the whale-fall community succession of [82], the Pisco Formation thus only records the sulphophilic stage, represented by sulphate-reducing bacteria (see also Bone preservation), and the reef stage, during which depleted bone remains are colonised by suspension feeders.

### Bone preservation

Vertebrate fossils across the three sequences show macroscopic and microscopic (both mineralogical and compositional) differences in bone preservation [38]. They are embedded in various rock types, from fine-grained to coarse-grained and scarcely cemented (diatomite, silt, sand, and volcanic ashes) to dolomite nodules filling or even surrounding the specimen [23]. At the macroscale, bones exhibit different colours (dark amber, red, white/pinkish, and white/grey-white) and degrees of hardness (fragile to very hard) [38]. Colour differences may be explained by variabilities in mineralogy, texture, and chemical composition, whereas different hardness values can be related to diverse degrees of mineralisation of the bone tissue, permineralisation (mineral filling of the bone porosity), and mineralogy. Crucially, bones from the same sequence (and at times from the same specimen) often display contrasting features that reflect very local conditions (e.g. the nature of the entombing sediment, oxygen availability, or organic matter supply) rather than macroscopic taphonomic traits such as the articulation and completeness degrees.

**Fig 19:**
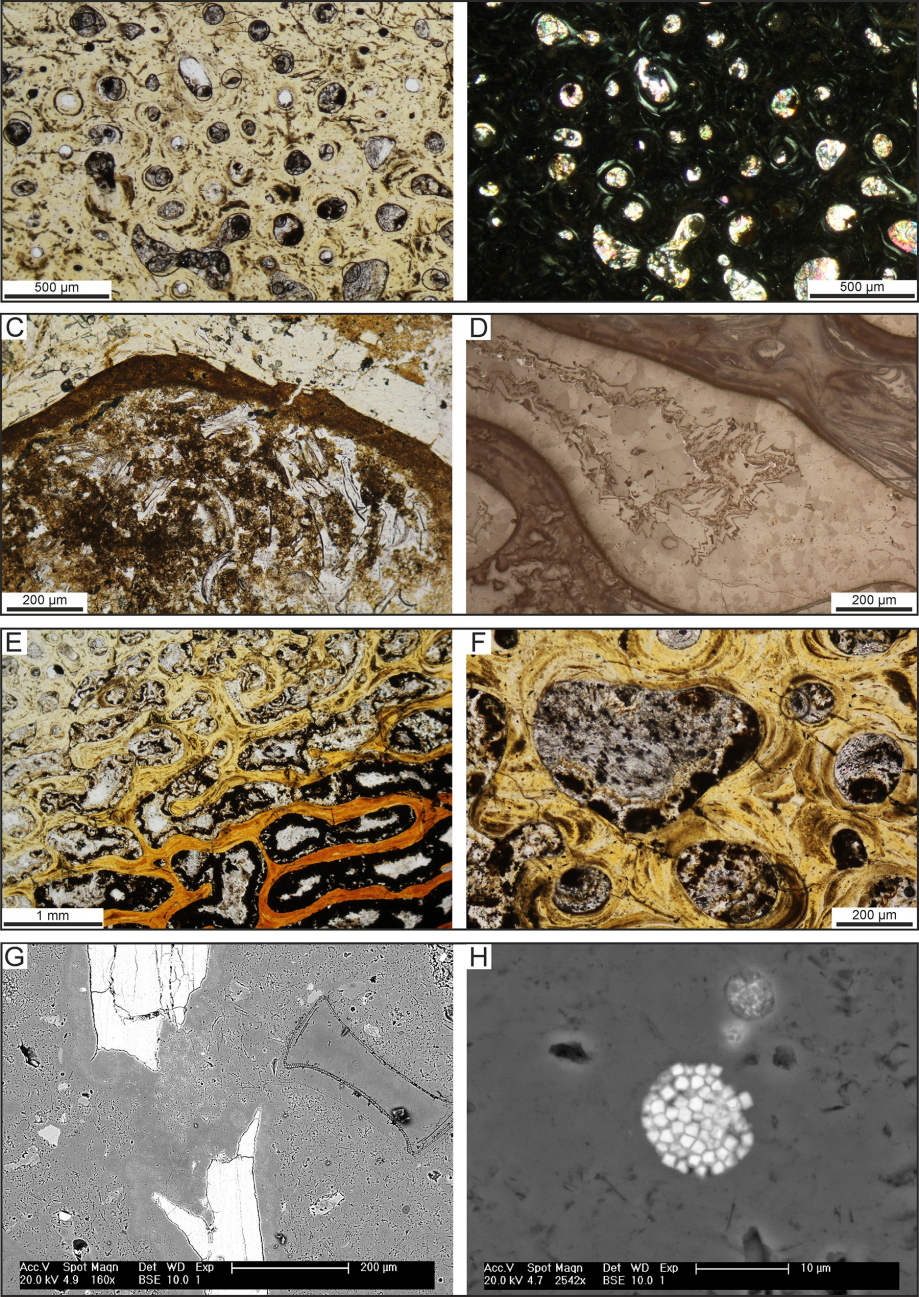
Modes of bone preservation. Photomicrographs and SEM images of fossil bones. A,B) Compact and cancellous bone of CCM11, skeleton of Cetotheriidae indet. from P1 diatomaceous silts preserved at Cerro Colorado, under transmitted plane-polarised (A) and cross-polarised (B) light, showing the preserved original microstructure (i.e., primary and secondary osteons, lamellae and osteocyte lacunae). This sample belongs to the type 2 of Bosio et al. [24]. C) Close-up of a trabecula in the cancellous bone of CLQM3, skeleton of Balaenopteridae indet. from P2 diatomites exposed at Cerro Los Quesos East, under transmitted plane-polarised light. This sample belongs to the type 1b of Bosio et al. [24]. Note the diatom-rich sediment infill and the cryptocristalline dolomite nucleated on the trabecula before the sediment filled the medullary cavity. D) Close-up of a dolomite-filled medullary cavity in the cancellous bone of CLQM1, skeleton of Balaenopteridae indet. from P2 diatomites exposed at Cerro Los Quesos East, under reflected light. This sample belongs to the type 1c of Bosio et al. [24]. Note the large size of the dolomite crystals and the evident zoning in composition in the drusy dolomite. E,F) Cancellous bone of the specimen CCM11, showing the bright red colour of the bone visible under the transmitted plane-polarised light getting more intense towards the centre. Note that the medullary cavities are filled by Fe-oxides that similarly increase towards the centre. Fe-oxides can fill the whole cavity or exhibit secondary filling in the centre. G) SEM-BSE image of a broken trabecula rimmed by dolomite, filling also a diatom frustule, in the cancellous bone of CLQM1. H) Close-up (SEM-BSE) of a framboidal Fe-oxide, ghost of a pyrite framboid, in the dolomite cement embedding and filling the bone cavities of CLQM1. Panel H after Gariboldi et al. [23].

Under the microscope, the original bone microstructure tends to be well preserved, showing primary and secondary osteons, osteocyte lacunae, and lamellae (Fig 19A and 19B). Outlined by fine opaque minerals (likely former iron sulphides), microcracks sometimes occur in a radial pattern surrounding osteons, but usually are not pervasive. Haversian canals and intertrabecular medullary cavities may be empty, partially filled by newly precipitated apatite, and/or occupied by sediment particles (Fig 19C), early diagenetic micro- and cryptocrystalline dolomite (Fig 19C and 19D), and late minerals such as gypsum/anhydrite (Fig 19E and 19F). Fe oxides also may line intertrabecular medullary cavities (Fig 19C and 19E–19G) and/or fill osteocyte lacunae and canaliculi as reported by Pfretzschner [101].

The presence of newly formed apatite in bone cavities suggests partial bone phosphatisation, with phosphorous being possibly provided by bioapatite dissolution or organic matter availability. Concentrations of tiny calcium phosphate crystals also frequently occur in the sediment immediately below specimens, indicating precipitation of phosphate minerals from porewater and, thus, conditions favouring apatite stability [39]. Dolomite associated with Fe oxides and ghosts of pyrite framboids (Fig 19H) implies conditions favouring carbonate precipitation. The latter may be induced by the presence of decaying organic matter, and locally result in the development of carbonate concretions surrounding the fossil [23,101]. Two distinct mechanisms of early diagenetic mineral formation are thus at work in the Pisco Formation and determine the final fossil preservation style: apatite dissolution-recrystallisation and dolomite precipitation. The latter may hamper the former, e.g. by reducing permeability during early diagenesis [38].

**Fig 20:**
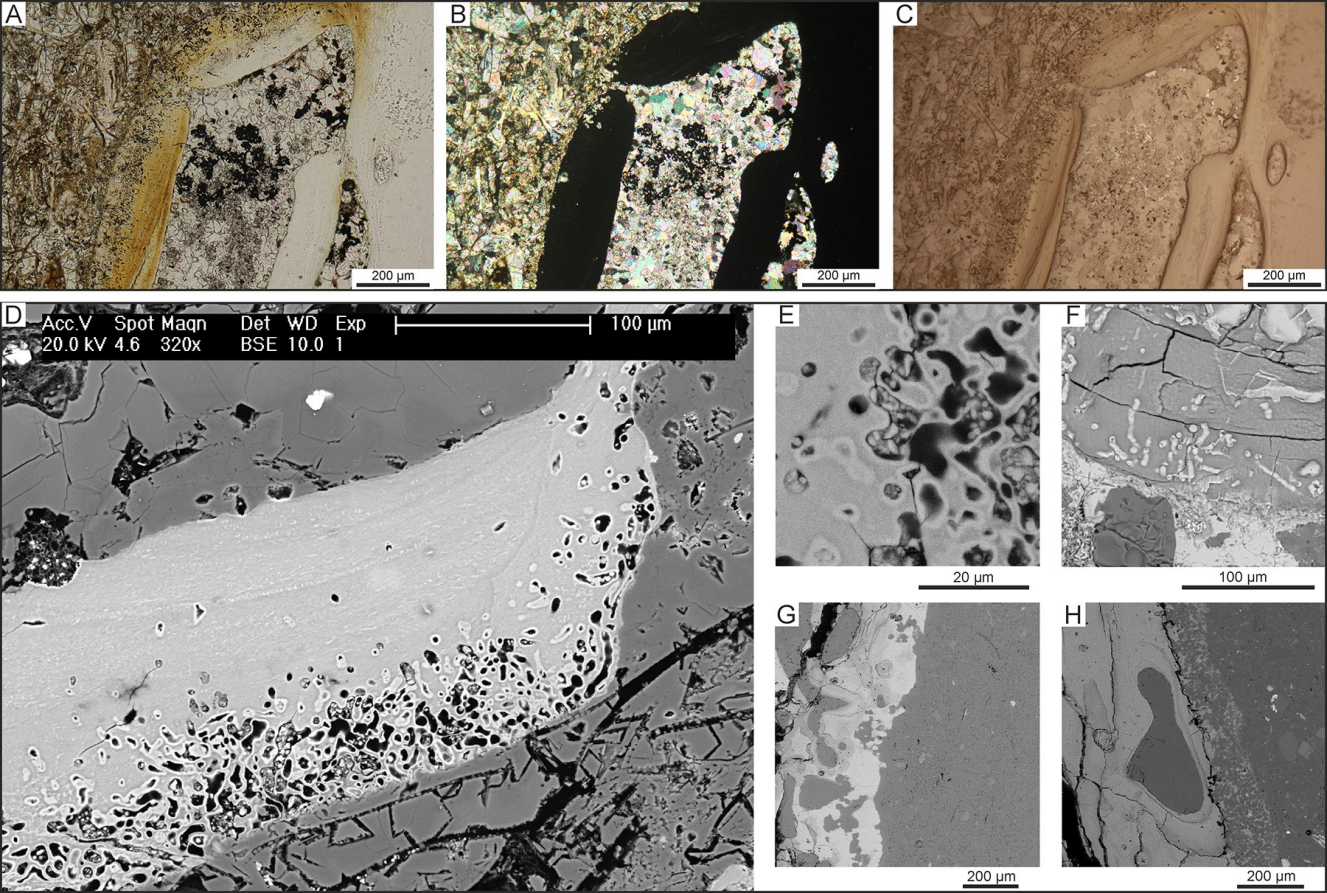
Bone microborings. Photomicrographs and SEM images of microborings affecting the external portion of the compact bone of fossil whale specimens. A, B, C) Microborings of the type B of Gariboldi et al. [23] that affect the bone tissue of CLQM10, skeleton of Balaenopteridae indet. from P2 diatomites exposed at Cerro Los Quesos East, under transmitted plane-polarised (A), cross-polarised (B) and reflected light (C). D, E) Close-ups (SEM-BSE images) of the microborings shown in panels A, B, C. Note the hypermineralised rim surrounding the borings that proved a bacterial origin. F) Microborings of the type A of Gariboldi et al. [23] that characterise the bone of CLQC67, skeleton of Cetacea indet. from P2 diatomites exposed at Cerro Los Quesos East (SEM-BSE image). G) Microborings of the type C of Gariboldi et al. [23] that affect the external compact bone of the specimen CLQM1, skeleton of Balaenopteridae indet. from P2 diatomites exposed at Cerro los Quesos East (SEM-BSE image). H) A new type of microborings, called type D, excavates and destroys the outer 150 μm of the compact bone of the specimen CLQC70, skeleton of Cetacea indet. from P2 diatomites exposed at Cerro Los Quesos East (SEM-BSE image). Panel A after Bosio et al. [24].

In general, bones from the Pisco Formation show none of the taphonomic features (e.g. *Osedax* borings) typically associated with whale-fall communities, which in turn matches the aforementioned scarcity of macro-invertebrates associated with the vertebrate fossils. In some cases, however, microborings are found within the first 200 μm of the outer part of the compact bone tissue [14, 23]. The resulting cavities can be empty or filled with sediment, apatite or diagenetic cements (Fig 20).

There are four types of microborings differing in dimensions and some other characteristics [34]. Type A is characterised by a diameter of ca 5 μm, a variable length with a maximum of 45 μm, and an infill of tardive gypsum also cementing the surrounding sediment (Fig 20F). Similar structures were described as ‘type 1’ by Danise et al. [79], and resemble the “Wedl type 2” boring of Jans [102], which have been attributed to fungi or cyanobacteria; however, they are neither bifurcated nor associated with a Haversian canal.

Microborings of type B are circular to slightly elongated, with a diameter of 3–10 μm and a length of 10–35 μm (Fig 20A–20E), thus recalling the so-called “linear longitudinal” Hackett tunnelling [102]. They may be empty or filled with apatite (Fig 20E), suggesting bone dissolution and recrystallisation as seen in bone cavities. A thick rim that appears brighter when imaged using BSE-SEM (backscatter scanning electron microscopy) and a stippled infill suggest bacterial activity [102], perhaps linked to a sulphophilic whale-fall community [82].

Microborings of type C have diameters of 15–35 μm, a length of ca 100 μm, frequently coalesce, and are filled with early diagenetic dolomite (Fig 20G). They resemble the Hackett tunnels of Jans [102] and may have been formed by fungi or cyanobacteria.

Finally, a new type of microboring, here named ‘type D’, is circular with a diameter of 5–20 μm, filled with early diagenetic dolomite, and so common that it nearly destroys the outer 150 μm of the cortical bone (Fig 20H). There is no thick rim appearing brighter under BSE-SEM and the borings were originally empty, perhaps suggesting the action of fungi and/or cyanobacteria [102].

### Baleen preservation

Instead of teeth, modern mysticetes bear comb-like, hair-fringed baleen plates that allow them to filter feed on vast amounts of small prey. Baleen is a soft tissue only moderately mineralised by crystallites of hydroxyapatite within an easily degradable keratinous matrix [103, 104] and as such rarely fossilises. To date, it has only been reported from a few Neogene localities worldwide, and is probably most abundant in the Pisco Formation [12, 13, 25, 26], which thus stands out as a *bona fide* Konservat-Lagerstätte (*sensu* Seilacher [22]).

**Fig 21:**
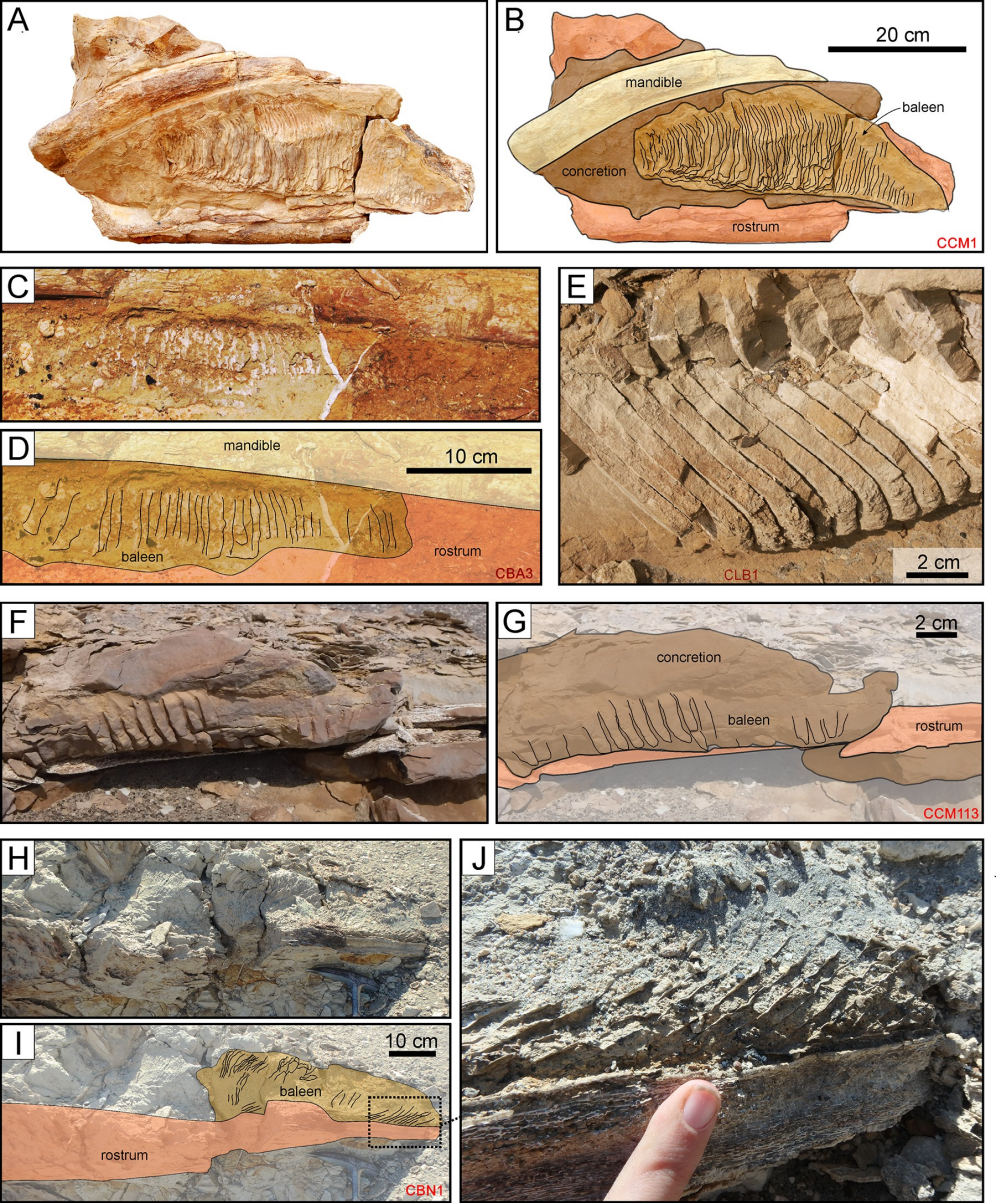
Modes of baleen preservation. Examples of baleen preservation in mysticete skeletons from various localities and horizons of the Pisco Formation. A, B) Field photograph (A) and corresponding line drawing (B) of the partial skull and mandible of CCM1, skeleton of Balaenopteroidea indet. from P1 diatomaceous silts exposed at Cerro Colorado, preserved in dorsal disposition and within a carbonate concretion (note that the figured specimen is part of a rolled boulder). The right baleen rack is preserved between the vomer and the right mandible. C, D) Field photograph (C) and corresponding line drawing (D) of the right baleen rack of CBA3, skeleton of *Piscobalaena nana* from P2 diatomaceous silts exposed at Cerro Ballena, preserved in ventral disposition in absence of any external concretion entombing the bones. E) Close-up of the preserved baleen of CLB1, skeleton of Balaenopteridae indet. from P2 sands exposed at Cerro la Bruja, preserved in ventral disposition and within a carbonate concretion. The right baleen rack is preserved between the right maxilla and mandible. F, G) Field photograph (F) and corresponding line drawing (G) of the right baleen rack of CCM113, skeleton of Cetotheriidae indet. from P1 silts exposed at Cerro Colorado, preserved in ventral disposition and within a carbonate concretion. H, I, J) Field photographs (H, J) and corresponding line drawing (G) of CBN1, skeleton of Mysticeti indet. from P2 diatomites exposed at Cerro Blanco North, preserved in ventral disposition in absence of any external concretion entombing the bones. Panel A after Gioncada et al. [25].

Esperante et al. [14] reported 70 whales preserving baleen (mostly *in situ*) from both diatomaceous and non-diatomaceous layer of the Pisco Formation. During our field activities, we observed only 7 such specimens: 4 balaenopterids and one cetotheriid from P2 (Fig 21A and 21B); and one balaenopteroid and one cetotheriid from P1 (Fig 21C–21J). So far, no cases of baleen preservation are known from P0.

Three of these baleen-bearing specimens have been investigated in detail and display fine-scale preservation of the baleen structures and tissues. Two articulated balaenopteroids from the upper diatomaceous mudstones of P1 exposed at Cerro Colorado (CLQM1; [25]) and the basal sands of P2 exposed at Cerro La Bruja (CLB1; [14]) preserve complete baleen racks inside dolomite concretions (Fig 21A, 21B and 21E). Fossilisation types include moulding of the baleen plates by dolomite-cemented sediment, as well as very fine-scale phosphatisation of the free terminations of the tubules (i.e. the baleen bristles). The original presence of hydroxyapatite crystallites likely helped to retard decay and facilitated mineralisation of the bristles. Both the latter and diatom frustules trapped inside the concretion preserve their original shape, suggesting rapid mineralisation prior to compaction inside a soft, organic-rich sediment prone to rapid dolomite nucleation [25]. Ghosts of iron sulphide framboids (now iron oxides/hydroxides) furthermore suggest the activity of sulphate-reducing bacteria in the whale carcasses and the immediate surroundings, which would have produced the alkaline environment required for both dolomite precipitation and phosphatisation [25].

The third specimen (CBA3; see also Shark bite marks) was found at Cerro Ballena (P2), and consists of an articulated skeleton of the cetotheriid *Piscobalaena nana* [26]. The baleen apparatus of this fossil (Fig 21C and 21D), which represents an entirely extinct morphotype, shows cellular-level mineralisation of the keratinous plates in the form of large (1–10 μm-sized) crystals of fluorapatite. The development of the latter was likely facilitated by high phosphorous concentrations in the porewater coupled with the presence of permeable, unconsolidated sediment. Together, these conditions allowed microbially-induced pH modifications near the decomposing carcass to inhibit dolomite formation and drive phosphatisation for a prolonged period of time [26]. As for the other two whales, the original presence of hydroxyapatite crystallites likely helped to retard decay during the early stages of mineralisation.

Our three case studies reveal two different preservation styles that nonetheless share two important features: a dependence on microbial activity associated with the decay of organic matter, and a certain predisposition of baleen to mineralise, likely thanks to the original presence of hydroxyapatite crystallites that may help to stabilise the otherwise keratinous plates and/or bristles.

### Digestive tract contents

Fossilised digestive tract contents (from the stomach, guts or regurgitations) provide rare but exceptionally informative insights into ancient trophic relationships [105]. Ingested prey undergoes rapid mechanical and chemical degradation during early digestion, and as such, it rarely fossilises. Even where preserved, it can only be unambiguously identified if its predator is itself exceptionally well preserved.

**Fig 22:**
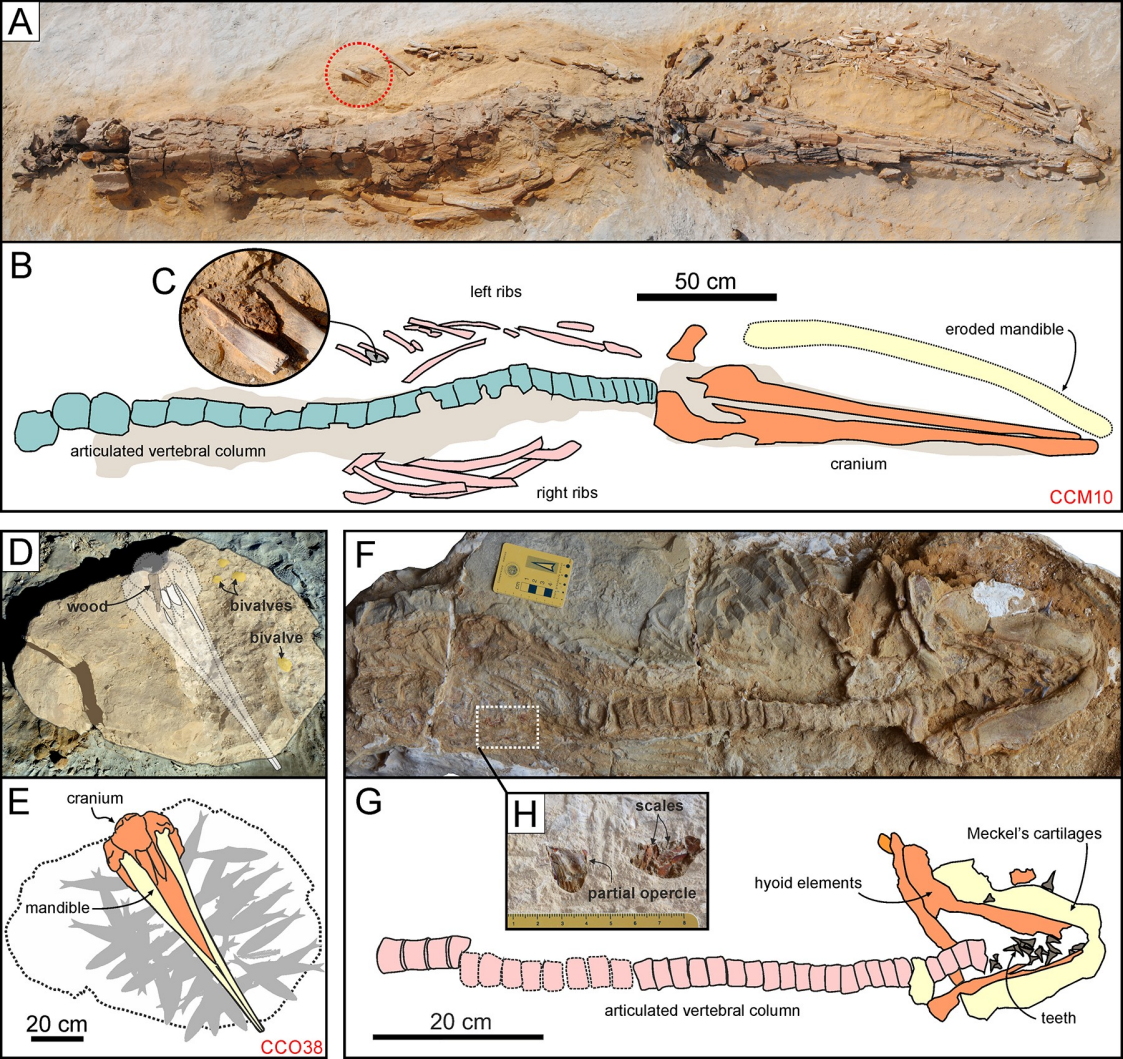
Digestive tract contents. Examples of preservation of digestive track contents in marine vertebrate skeletons from various localities and horizons of the Pisco Formation. A, B, C) Field photographs (A, C) and explanatory line drawing (B) of CCM1, skeleton of Cetotheriidae indet. from P1 diatomites exposed at Cerro Colorado, preserved in dorsal disposition and exhibiting a dense aggregate of skeletal and dermal fish remains, here interpreted as comprising a fossilised stomach content, in-between the left posterior ribs (C). D, E) Field photograph (D) and explanatory line drawing (E) of CCO38, skeleton of *Messapicetus gregarius* from P1 diatomites exposed at Cerro Colorado, preserved in ventral disposition and exhibiting an accumulation of skeletons of *Sardinops* cf. *sagax*, here interpreted as comprising a fossilised regurgitalite, in close proximity of the skull. F, G, H) Photograph (F) and explanatory line drawings (G, H) of CPI-7899, skeleton of *Cosmopolitodus hastalis* from undifferentiated Pisco strata exposed at Cerro Yesera, preserved in ventral disposition and exhibiting a dense aggregate of skeletal and dermal fish remains, here interpreted as comprising a fossilised stomach content, in the abdominal region (H). Panel D modified after Lambert et al. [30]; panel F modified after Collareta et al. [111].

Among cetaceans, fossilised digestive tract contents are largely limited to basilosaurid archaeocetes [106–109]. For neocetes, the only examples worldwide come from the P1 strata exposed at Cerro Colorado (an observation that further substantiates the Konservat-Lagerstätte status of the Pisco Formation). They include a dense aggregate of skeletal and dermal fish remains from CCM10, an indeterminate cetotheriid partly encased in a dolomite concretion (Fig 22A–22C; [28]). Most of the aggregate consists of Pacific sardines (*Sardinops sagax*), small epipelagic schooling fish that are a key component of the modern Humboldt Current System [110]. The architecture, preservation and location of the aggregate between the posterior left ribs of the host whale suggest that it comes from the cetacean forestomach [28]. A similar aggregate was recently discovered in CCM11, which belongs to the same species as CCM10 (Fig 7A and 7B). Here reported for the first time, this aggregate also includes sardine skeletal elements, and was found posterior to the rib cage; as such, it might derive from either the gut or the stomach.

Numerous sardines (40–60 articulated skeletons) also occur in close association with CCP38, a specimen of the stem ziphiid *Messapicetus gregarius* preserved within a well-developed dolomite concretion [30]. Based on the position of the fish specimens around the head of the whale, their similar size and the absence of digestion marks, it seems plausible that they originated from a single school and were regurgitated–perhaps because of toxin poisoning–shortly after capture.

Identifying the digestive tract contents of sharks is further hampered by the cartilaginous nature of their skeleton, and the attendant rarity of articulated fossil specimens. Even so, a well-preserved example was recovered from undifferentiated Pisco strata exposed at Cerro Yesera, in the form of a partial skeleton of a juvenile *Cosmopolitodus hastalis* preserving a cluster of fish remains (including several scales and an opercle of *Sardinops* cf. *sagax*) in its abdominal region [111].

Overall, the surprisingly varied digestive tract contents from the Pisco Formation represent an outstanding example of exceptional preservation and help qualify the unit as a true Konservat-Lagerstätte. Together, they evoke a food web built on small, epipelagic schooling fish resembling the modern Humboldt Current System. Here, high levels of primary productivity sustain abundant populations of anchovies (*Engraulis ringens*) and sardines (*Sardinops sagax*), which in turn serve as food for a plethora of bony fishes, sharks, marine mammals, and seabirds [110, 112].

### Carbonate concretions

**Fig 23:**
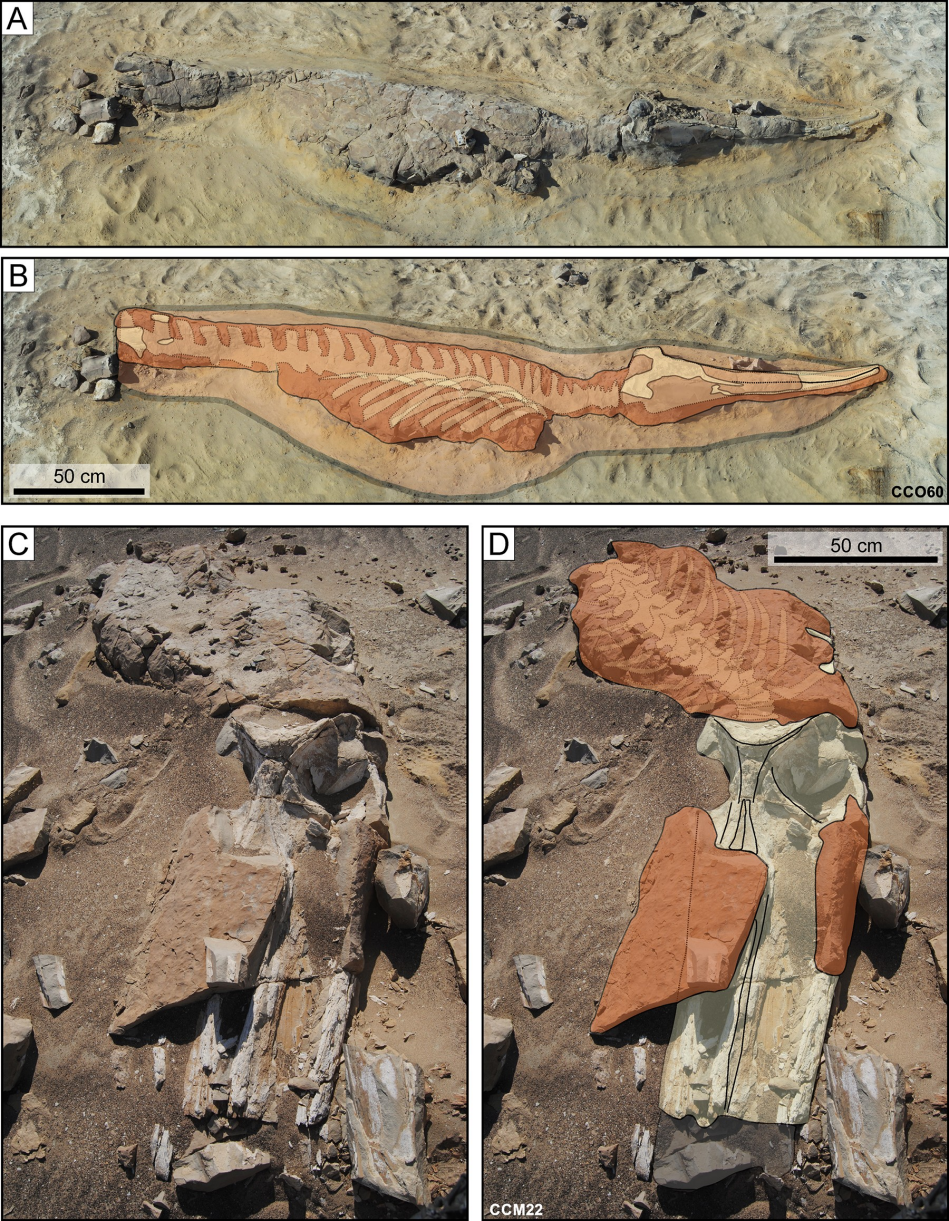
Fossil vertebrates enclosed within carbonate concretions. Carbonate concretions enclosing marine vertebrate skeletons from the P1 strata of the Pisco Formation exposed at Cerro Colorado. A, B) Field photograph (A) and corresponding line drawing (B) of CCO60, skeleton of *Messapicetus gregarius* preserved in silts, in lateral disposition, exhibiting high degrees of articulation and completeness (4,4), and a conspicuous external carbonate concretion. Note that this specimen appears as preserved within a YBR sequence (yellow-black-red sediments of Gariboldi et al. [23]. C, D) Field photograph (C) and corresponding line drawing (D) of CCM22, skeleton of Cetotheriidae indet. preserved in sands, in dorsal disposition, exhibiting high degrees of articulation and completeness (4,4), and a conspicuous external carbonate concretion. Around the skull, this concretion underwent widespread erosion in the present-day desert environment.

**Fig 24:**
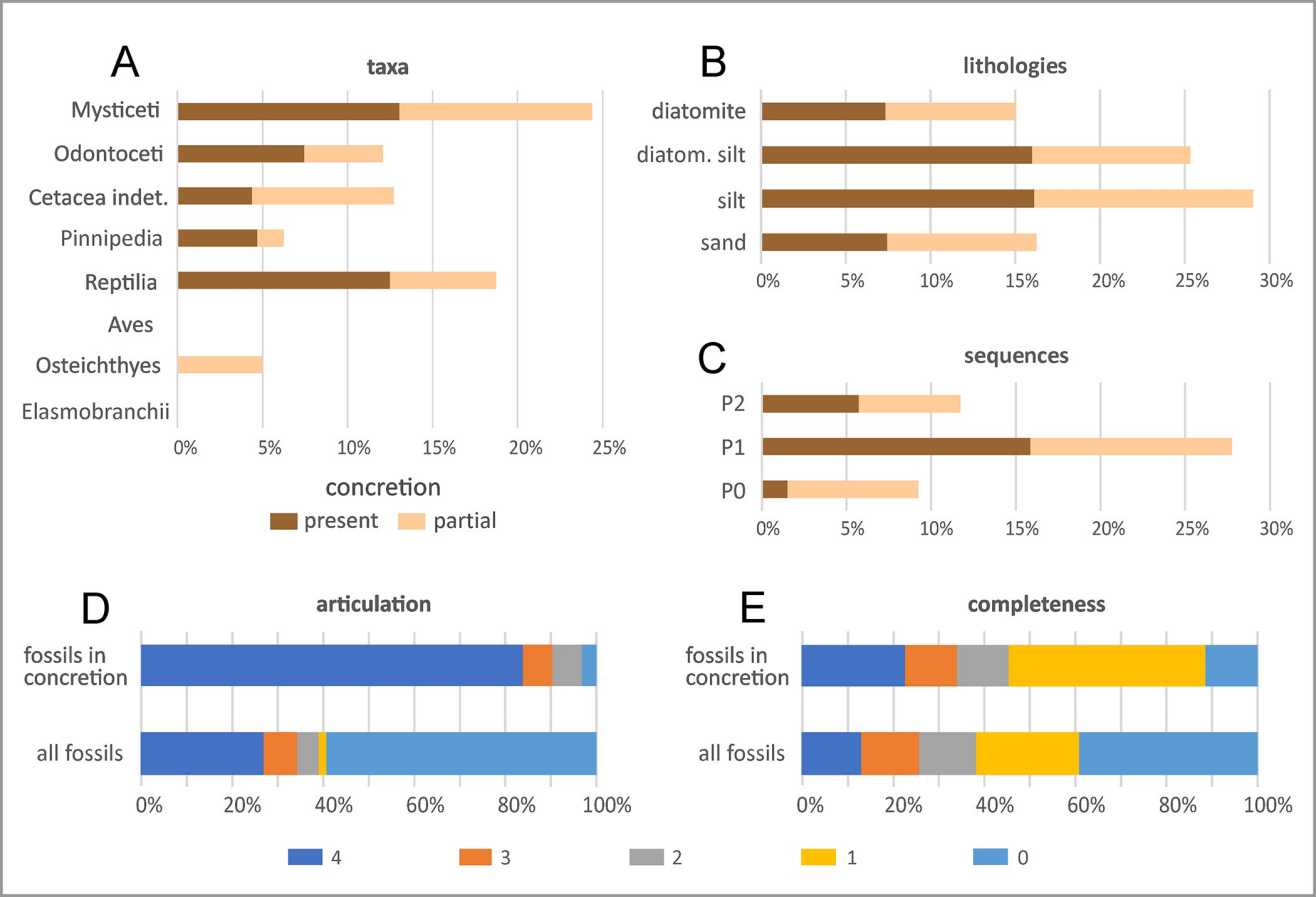
Distribution of carbonate concretions. Bar diagrams showing the distribution of carbonate concretions enclosing fossil marine vertebrates through the main taxonomic groups (A), the main lithologies (B), and the three sequences (C) of the Pisco Formation in the study area. The bar diagrams at the bottom of the figure highlight the different degrees of articulation (D) and completeness (E) displayed by fossil vertebrates preserved in concretions compared to the total vertebrate assemblage.

Dolomite and, less commonly, calcite nodules may totally or partially enclose vertebrate skeletons (Fig 23). Size appears to be important, with larger specimens like mysticetes being more likely to induce carbonate precipitation (Fig 24A; [23, 39]). This may be explained by their higher amount of bone lipids in soft matter and bone tissues, whose degradation plays a pivotal role in triggering nodule formation [23]. Low permeability around the carcass is another facilitating factor [39], and may account for the high number of concretions associated with marine reptiles protected by a shell (turtles) or armour (crocodilians), which would limit access to oxygenated waters and induce various reduction processes, including sulphate reduction (Fig 24A). Similarly, cetacean brain cavities are often the location of dolomite nodules [39].

In term of their distribution across different lithologies, the abundance of nodules appears to be controlled by a complex interplay of sediment permeability (highest in sands), sediment ability to ‘engulf’ carcasses (highest in the diatom-rich deposits), the distribution of mysticetes across the different lithologies (highest in diatomites), and the distribution of different lithologies and taxa across the three allomembers (Fig 24B and 24C).

Specimens in nodules tend to be well articulated, but they are not as complete as one might expect (Fig 24D and 24E). This supports the notion that loss of completeness primarily occurs while a carcass is floating, whereas disarticulation happens mostly on the seafloor. Nodule formation depends on rapid burial of the carcass [23, 25], which in turn leaves little room for disarticulation. Once formed, carbonate nodules protect specimens from disarticulation through further soft-tissue decay [113] and/or diagenetic compaction [114]. Specimens in nodules do also show higher levels of completeness, albeit marginally so. This may be explained by the fact that organic matter is required for nodule formation [115], which in turn favours more complete skeletons with larger quantities of soft tissue.

The mechanism of dolomite formation can be summarised as follows. Following the arrival of a carcass on the seafloor, low oxygen levels quickly inhibit aerobic degradation of larger carcasses, especially if they are rapidly buried in fine-grained sediment [116]. Starting from the interior of the carcass, anaerobic decay of bone lipids and, eventually, organic matter dispersed in the sediment around the carcass leads to bacterial reduction of Mn, nitrates and Fe, as well as to sulphate reduction and methanogenesis. The resulting increase in alkalinity and depletion of dissolved sulphate (the latter normally inhibiting dolomite formation in seawater; [117]) rapidly (i.e. typically within weeks; [118]) create conditions favouring the precipitation of a finely crystalline carbonate cement within the bone cavities and surrounding sediment, thus forming a ‘concretion’ [23]. As evidence of its microbial origin, the latter often contains framboids of iron oxides arising as a by-product of sulphate reduction.

The dolomite concretion, in turn, protects the specimen by (i) forming a physical and chemical barrier between it and the surrounding sediment, benthos, and porewater; (ii) infilling bone cavities, which counteracts compaction; (iii) preventing further disarticulation; and (iv) shielding the fossil against weathering following exhumation in the present-day subaerial environment [23, 25, 39]. The preservation of fossils enclosed in dolomite nodules is accordingly very good, and can even include soft tissues like baleen (see Baleen preservation).

### Inferred mechanisms of rapid burial

Marine vertebrate fossils are extremely abundant in the Pisco Formation [17, 18, 35] and show a range of preservational styles, including complete and/or articulated skeletons occasionally associated with soft tissues and digestive tract contents [13, 25, 28].

Previous studies attributed this exquisite preservation to a variety of factors, such as rapid burial of specimens in a soupy substrate, high background or episodic (high-energy) sedimentation rates, and early diagenetic processes [12, 13, 23, 26, 119]. A better understanding of the processes driving rapid burial may be encoded in the relationship between fossils and associated sedimentary structures. To unravel these, we examined representative specimens at three key localities.

#### Cerro Colorado and Cerro los Quesos

We examined two articulated balaenopteroids embedded in a rather monotonous succession of finely laminated white diatomites interbedded with sparse nodules, volcanic ash-fall deposits, and dolomite-cemented beds [71, 120]. As a whole, the succession contains numerous vertebrate skeletons with varying levels of articulation and completeness [17, 18]. Grain size and sedimentary structures suggest deposition by suspension settling of diatom frustules in a low-energy offshore setting, i.e. below storm-wave base.

**Fig 25:**
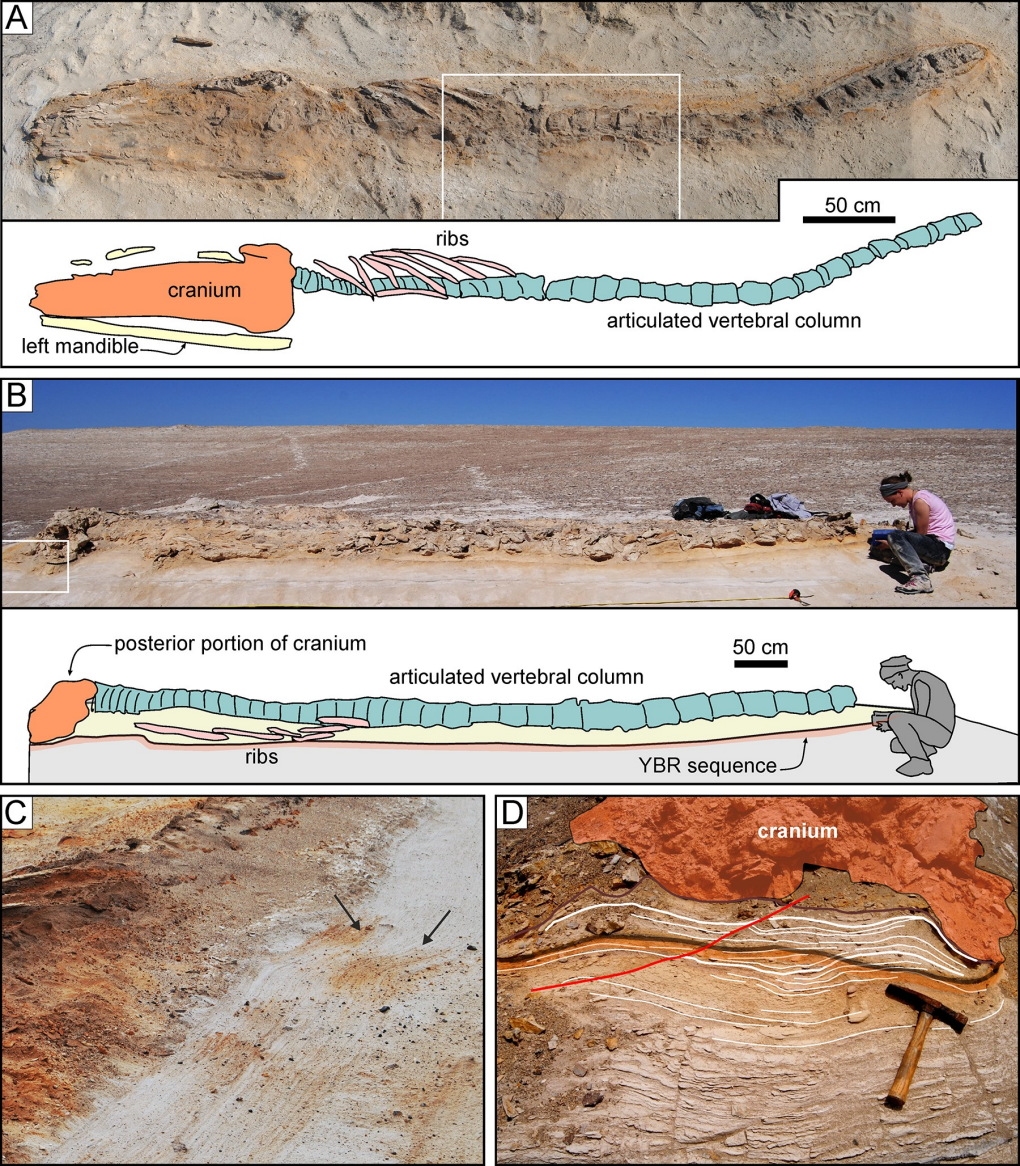
Soft-sediment deformation structures. Soft-sediment deformation structures associated with vertebrate skeletons from various localities and horizons of the Pisco Formation. These deformation features are interpreted to have formed around vertebrate carcasses that partially sank into the sediment. Note the presence of a yellow-black-red (YBR) sequence around both skeletons. A, B) Field photograph (A) and corresponding line drawing (B) of CCM28, skeleton of Cetotheriidae indet. from P1 diatomaceous silts exposed at Cerro Colorado, preserved in ventral disposition, exhibiting a high degree of articulation and a moderately high degree of completeness (4,3). The white box in panel A indicates the area detailed in panel E. C, D) Field photograph (C) and corresponding line drawing (D) of specimen CLQM58, skeleton of Mysticeti indet. from P2 diatomites exposed at Cerro los Quesos East, preserved in dorsal disposition, exhibiting high degrees of articulation and completeness (4,4). The dorsal disposition and articulation and completeness values of CLQM58 suggest it sank back-first onto the seafloor, kicking up soupy sediment that engulfed it. E) Close up of the deformed diatomaceous silt laminae around CCM28. Arrows point to deformed laminae gently dipping towards the skeleton. F) Detail of the distorted diatomite laminae underlying the cranium of CLQM58 (cross section through the entombing sediment). Note the YBR (yellow-black-red) sequence passing across both the deformed laminae (white lines) and a small-scale fault (red line) indicating that soft-sediment deformation occurred shortly after deposition and prior to the establishment of the early diagenetic reduced conditions induced by carcasses degradation. Panel C after Gariboldi et al. [23].

Around the fossil skeletons (Fig 25A–25D) the finely laminated sediment is notably disturbed and deformed (Fig 25E and 25F) and, locally, truncated and displaced by small-scale synsedimentary faults (Fig 25F). In addition, fossils are surrounded by a characteristic sequence of centimetre-scale coloured bands that are arranged in a regular yellow-black-red sequence and cut across the deformed and faulted laminae (Fig 25F). These bands clearly postdate sediment deformation, and suggest redox-sensitive element enrichment (Mn, Fe) in the reducing conditions created by degradation of the whale carcass during early diagenesis [23].

#### Las Antenas

Sediments at this locality comprise variable mixtures of diatoms, silt, volcanic ash, and fine-grained sand, and are characterised by predominant hummocky cross-stratification and sporadic ripple cross-lamination. They are variably intercalated with stringers and lenses of small pebbles, U- and V-shaped gutter casts a few decimetres across and several centimetres deep, and sand beds and volcanic ash layers a few centimetres thick. This combination of primary sedimentary structures and grain sizes suggests deposition in the offshore transition zone (ranging from storm-wave base to fair-weather wave base), as well as frequent, high energy disturbances at the sediment-water interface by unidirectional and oscillatory flows generated under severe storm conditions [121–123].

**Fig 26:**
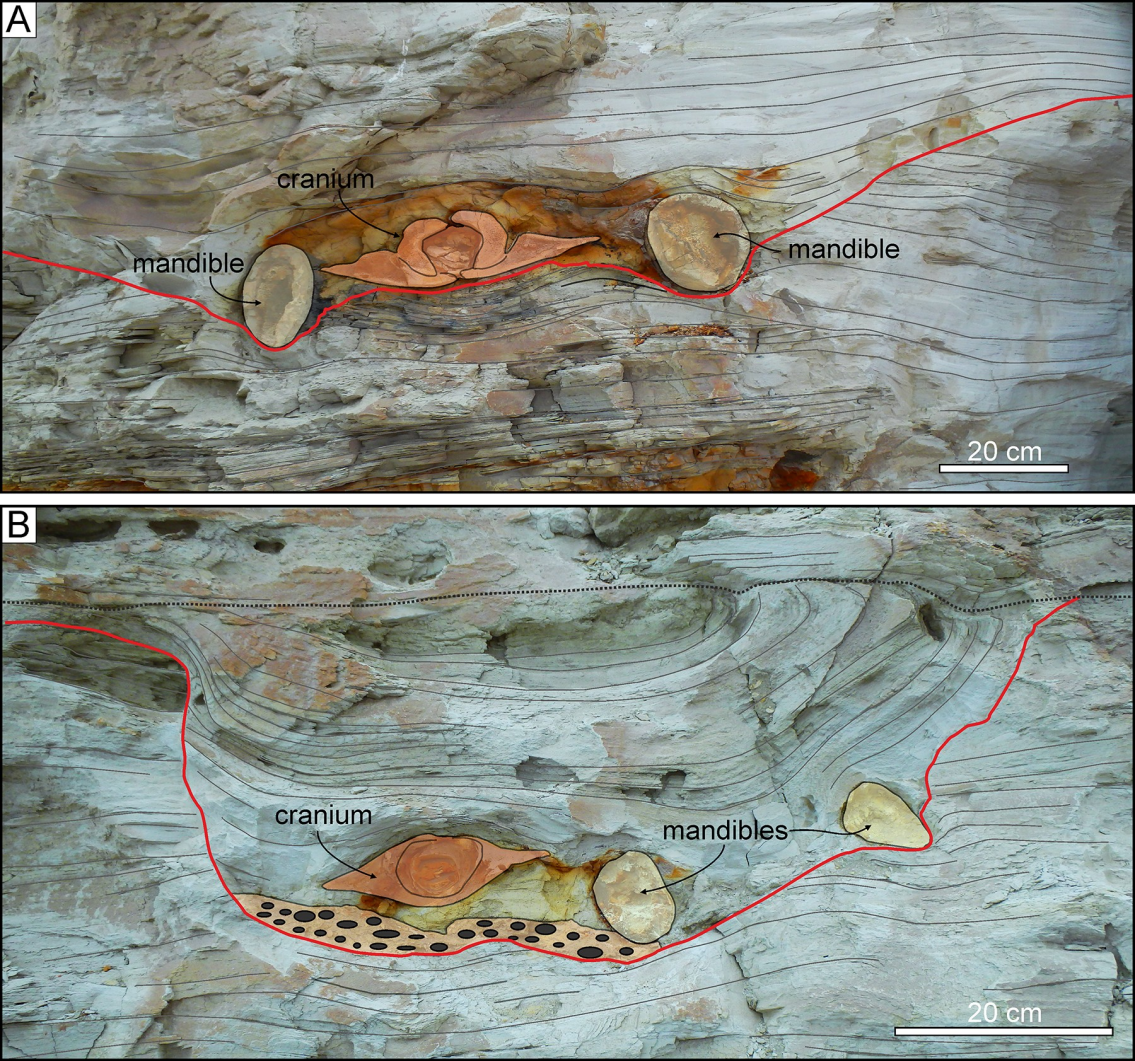
Scour structures. Field photographs of two distinct scour structures associated with mysticete skeletons from undifferentiated Pisco strata exposed at Las Antenas. Each scour preserves the cranium and mandibles of an indeterminate mysticete. In both cases the specimen is preserved in dorsal disposition and crops out in a transverse vertical section, resting directly on a distinct erosion surface (bold red line). The cross-sectional shape of these scours varies from asymmetrical to symmetrical. A) Close up of a symmetrical scour displaying margins that widen and fade upwards. Note the deformation produced during compaction by the cranium and mandibles protruding into the underlying sediment. The geometric relationships between the transverse sections of the cranium and mandibles suggests that the bones more or less retain their anatomical disposition. B) Close up of an asymmetrical scour displaying a steep left-hand margin and a gentler right-hand margin. The steep margins imply rapid deposition and fill following the formation of the scour. The geometric relationships between the transverse sections of the cranium and mandibles suggests that the latter were significantly displaced from their anatomical position. Note the right-side mandible partially protruding into the underlying sediment and the conspicuous internal deformation of the scour fill laminae, with those at the right-hand side of the scour being rotated to vertical or folded and truncated in the upper part by a horizontal erosion surface (black dotted line).

The two cetacean skeletons documented at this site (Fig 26) are exposed along vertical outcrop faces and sectioned through their heads. In cross section, each of them sits inside a flat-bottomed depression of nearly the same size (about 80 cm wide and 50 cm deep) as the whale itself. The walls of these structures truncate, but do not deform, the laminae of the surrounding diatomaceous silt, suggesting that the depressions themselves were formed by erosion. Their three-dimensional geometry is obscured by the almost vertical nature of the outcrop, but excavations along irregular cliff walls suggest them to be spoon-shaped, rather than linear features.

The specimen in Fig 26A lies in the deepest part of a symmetrical scour with margins that flare upwards. The exposed bones, including a cranium and two mandibles roughly in anatomical position, are completely enclosed in a dolomite concretion. They protrude a few centimetres into the underlying sediment, deforming the laminae immediately beneath them. The scour-filling sediment consists of laminated diatomaceous silts resembling the surrounding deposits. Its laminae initially drape over the shape of the underlying fossil and the walls of the depression, but then gradually become less concave as the scour is filled.

The specimen in Fig 26B comprises a cranium and two mandibles inside an asymmetrical scour with one vertical and one sloping wall. The cranium and one of the mandibles are encased in a dolomite and lie atop a lens of small, well-rounded pebbles set within a matrix of medium- to coarse-grained sand at the bottom of the depression. The other mandible is displaced relative to the skull and partly protrudes into the underlying sediment. The scour is filled with sagging diatomaceous silt laminae that thin and onlap on to the left-hand wall, drape the fossil remains in the centre of the depression and become oversteepened to overturned on its right-hand side, where they are deformed into a sharp-hinged recumbent fold (Fig 26B). At the top, the laminae are sharply truncated by undisturbed layers of similar lithology.

#### Mechanisms of rapid burial

Exceptional preservation of vertebrate fossils on the seafloor crucially depends on rapid burial, but the mechanisms behind this phenomenon have so far remained largely obscure. Unusually high background sedimentation rates may provide an obvious explanation, but this is not supported by evidence on the ground. Thus, average net sedimentation in the Pisco Formation has been estimated at just 32 cm/ka [35], a rate at which it would have taken several thousand years to cover a large carcass. Such a prolonged period of exposure is inconsistent with fossil preservation, exceptional or otherwise [124], and suggests that a different burial process must have been at work.

Although there is currently no obvious evidence from modern examples and/or experimental taphonomy to elucidate this question, laboratory and field experiments investigating the burial of bivalve shells [125], cylindrical sea mines [126–133], and shipwrecks [134, 135] provide suitable analogues. Results from these studies show that rapid burial in shallow-water settings may arise from impact burial and scour-induced self-burial [136]. Impact burial occurs when an object sinks at a velocity high enough to cause partial or complete burial upon impact into extremely soft or soupy substrates. By contrast, scour-induced self-burial results from local perturbation of wave and current-related flow patterns around an obstacle sitting on an erodible seafloor.

Self-burial starts when a stationary object on the seafloor causes a local increase in flow velocity and turbulence. This results in a higher sediment transport capacity and promotes the development of a scour pit wrapping laterally around the obstacle [137]. With time, ongoing removal of sediment may undercut the object and cause it to fall to the very bottom of the pit. Once the object settles below the level of the surrounding seafloor and no longer obstructs flow, sediment begins to fill the depression [125]. Depending on the conditions, a scour can bury a 2 m long and 0.5 m wide sea mine within days or weeks [127], with the process being most effective during severe storm events [130, 131]. In addition, a lag of coarse-grained sand and pebble-grade gravel may develop at both ends of the mine as finer sediment is gradually winnowed away [126, 129].

At Cerro Colorado and Cerro Los Quesos, the occurrence of soft-sediment deformation of the laminae immediately below and around the skeletons suggests that the sinking carcasses penetrated a soft or soupy sediment. This kind of instant impact burial likely enhances the chances of exceptional preservation [138], but, depending on sediment properties, need not always cover the entire skeleton. Any exposed parts are still subject to degradation and disarticulation, resulting in partial preservation and/or subsequent self-burial (see below).

At Las Antenas, vertebrate fossils occur within spoon-shaped hollows surrounded by sediments documenting recurring high-energy storm events. A previous study by [139] interpreted these hollows as linear channels carved by storm-related, offshore-directed unidirectional flows resulting from a combination of denser, sediment-laden runoff from the coast and currents secondary to storm surges. In this scenario, the occurrence of skeletons inside the hollows would be largely accidental. Though plausible at first sight, this model fails to explain (i) why the scour pits and the skeletons inside them consistently show similar dimensions; (ii) why nearly all scour pits contain a vertebrate fossil; and (iii) why the grain size of the scour-filling material is similar to that of the surrounding sediment, rather than coarser as would be expected in case of strong offshore-directed, erosionally-confined unidirectional flows.

**Fig 27:**
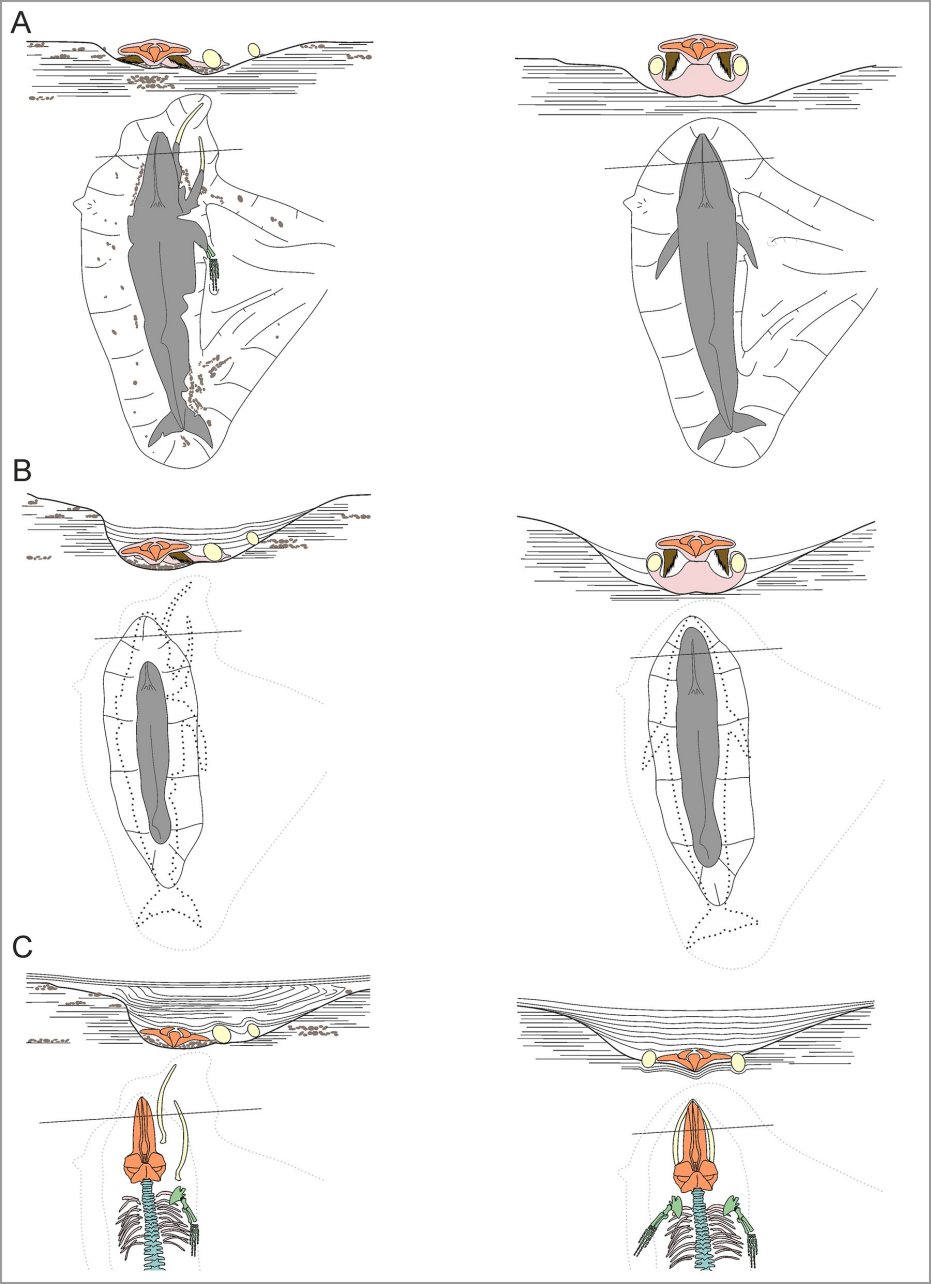
Proposed mechanism of rapid self-burial (I). Diagrams summarising steps in the proposed storm-related self-burial mechanism rapidly burying large cetacean carcasses resting on the seafloor at various stages of decay. Note the smaller scale in profile view. A) In this scenario the whale carcass reaches the sediment-water interface relatively soon after death, largely intact or in a very early stage of decay after a short floating phase at the sea surface. Under storm conditions, currents and waves develop a scour pit around the carcass. As erosion around and beneath the carcass proceeds and the scour depression deepens, the carcass gradually sinks into the deepening depression. Concentration of small pebbles near the head results from winnowing of loose fine-grained sand and silt, leaving gravel-size particles in the scour depression. B) Once the carcass has settled below the level of the surrounding bottom, where it no longer acts as an obstruction to flow, the scour depression is filled in by sediments, completely burying the carcass before significant decomposition may take place. C) Deformation and sagging of laminae in the scour-filling sediments were presumably due to decay of the soft tissues and compaction by the weight of overlying sediment, which resulted in some squashing of the sediments deposited inside the scour and partial penetration of fossil bones into the underlying sediments. Bone colours in the figure refer to those coded in Fig 3.

**Fig 28:**
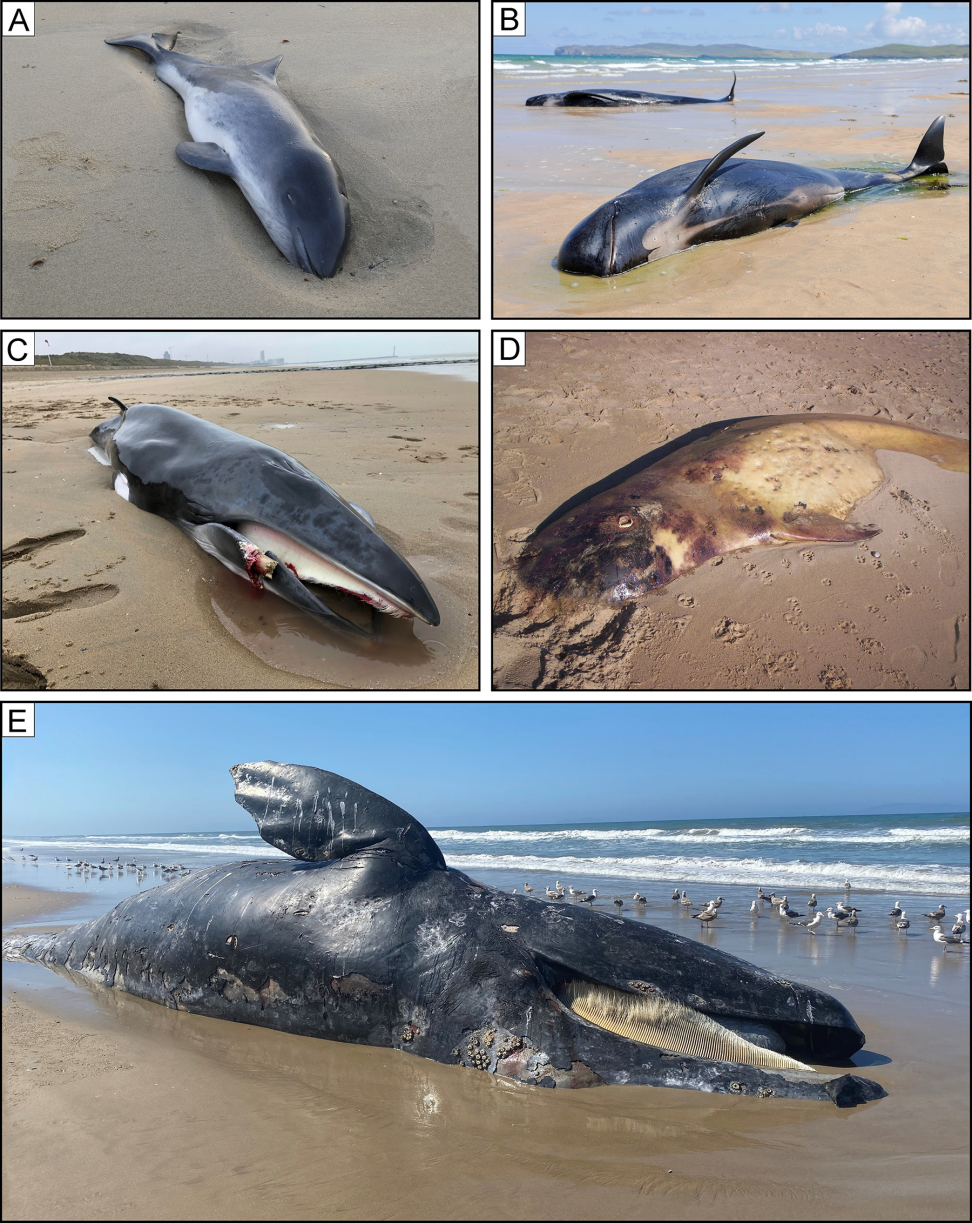
Proposed mechanism of rapid self-burial (II). Modern whales stranded and partially buried in the swash and intertidal zones of sandy beaches. All specimens sit in a scour depression developed around them as a result of erosion of the sediment surface by intense wave action. A) Specimen of *Phocoena phocoena* stranded in the Middelkerke beach (Belgium) in 2017 (photograph by J. Haelters). B) Two pilot whales (*Globicephala melas*) of 12 stranded in the Falcarragh beach (Donegal, Ireland) in July 2014 (photograph by S. Barnes). C) Specimen of *Balaenoptera acutorostrata* stranded in Bredene beach (Belgium) in December 2020 (photograph by J. Haelters). D) Carcass of *Ziphius cavirostris* found partially buried in the Eraclea Minoa beach (Sicily, Italy) (photograph by G. Insacco). E) A gray whale (*Eschrichtius robustus*) stranded at Emma Wood State Beach (Ventura County, California, USA‎) in May 2020 (photograph by A. Bautista).

Taken together, the available evidence suggests that scour pits are directly related to the fossils within them, and that scouring facilitated rapid burial and preservation of the Pisco marine vertebrates. During storms, oscillatory currents scoured the immediate surroundings of a carcass, until the latter destabilised and slid into the resulting depression. This was then followed by backfilling of the scour pit, thus completing the self-burial process (Fig 27). Similar mechanisms seemingly affect marine mammal carcasses in the swash zone of modern sandy beaches (Fig 28), where they rapidly become embedded in the shore sediments, where they rapidly become embedded in the shore sediments [63].

The slight disarticulation of the specimen in Fig 26B, also commonly observed elsewhere in the Pisco Formation [14], suggests partial loss of connectivity prior to deposition on the seafloor, perhaps during a relatively short floating phase [116]. Nevertheless, sedimentological features and preservation style suggest that both the analysed specimens from Las Antenas were buried within days or weeks after their death. The presence of a vertical depression wall implies rapid scouring and backfilling of the pit, perhaps during a single storm event. The backfilling laminae were bent downwards shortly after deposition, when the sediment was still unconsolidated, but before the deposition of the directly overlying undeformed strata. Their deformation likely reflects differential compaction as decay of the carcass progressed and the skeleton collapsed.

Additional, albeit indirect, evidence for rapid burial comes from the presence of a concretion, which probably formed in response to the ongoing decay of soft tissues soon after burial [25, 39, 140]. The mandible on the right in Fig 26B lacks dolomite and instead has sediment laminae draped directly over it, suggesting that most of the associated soft tissues had been lost or otherwise dispersed by the time the carcass was buried.

## Summary of the factors leading to the Lagerstätte formation

Both the abundance and quality of the fossil vertebrates from the Pisco Formation have long been noted, but it was not until the 2000s that the drivers behind their formation started to be systematically investigated. This work was pioneered by Brand et al. [12], who considered (i) anoxia at the water-sediment interface, (ii) covering of carcasses by diatom mats, and (iii) rapid burial as their main working hypotheses. Arguing that modern depositional environments and actualistic taphonomic models cannot fully explain the Pisco Lagerstätte, they settled on rapid burial, fast enough to cover large-sized mysticete carcasses within few weeks or months, as the most likely explanation. Such rapid burial would imply diatom accumulation rates at least 3–4 orders of magnitude faster than today, i.e. centimetres per week or per month, rather than per thousand years [12].

This hypothesis was reiterated by subsequent studies [13, 14, 34], one of which, however, suggested accumulation rates of just 11–30 cm/1000 years [14]. These values are consistent with subsequent estimates from Cerro Los Quesos and other productive basins worldwide [35]. As such, they strongly argue against rapid sediment accumulation as a driver of fossil preservation [35, 141], although abrupt events like seasonal diatom dumps, hyperpycnal flows, and submarine slides may still at times have played a role.

Below, we summarise the palaeoenvironmental conditions and processes that may have driven the formation of the Pisco Lagerstätte, based on the results of our multi-year research programme. In doing so, we specifically account for the different preservation styles that may coexists at some sites, yet characterise particular layers and lithologies at others.

### Life

Lagerstätten are exceptional death assemblages, but, at least in some instance, also record an original abundance of life [142]. Assuming constant environmental conditions (e.g. sedimentation rates, sea bottom conditions, scavenging activity), the abundance of fossils at a particular site should roughly reflect the number of animals that once frequented it. If so, then the rich marine vertebrate assemblages of P1 and P2 hint at a diverse and abundant late Neogene fauna (see also Taxonomic distribution). Some forms, like the cetaceans *Messapicetus* and *Brachydelphis*, may have inhabited the East Pisco Basin for much of the year, using it both as a feeding and as a breeding/calving ground [142]. Likewise, the copper shark *Carcharhinus brachyurus* and, perhaps, other elasmobranchs used this area as a nursery ground, giving rise to an unusually high concentration of juvenile sharks and rays [21]. This intense (and diversified) utilisation of the East Pisco Basin is likely reflected in the large number of fossils it has yielded, which even by its sheer size raises the chances of exceptional preservation.

### Death

Harmful algal blooms are a well-known cause of mass mortalities among fishes, seabirds, and marine mammals [143–145], and are thought to explain high concentrations of vertebrate fossils in some Neogene marine deposits [2, 146]. The Pisco Formation records extensive algal blooms in the form of thick packages of diatomaceous mudstones [12–14, 19, 33, 35, 42, 52, 65, 120], and catastrophic mass mortality events have been invoked to explain batches of aligned cetacean skeletons at Cerro Los Quesos [17]. Further, the death of a specimen of *Messapicetus* (see Digestive tract contents) preserved alongside regurgitated sardines has specifically been attributed to possible poisoning by algal toxins [30], which is in line with sardines being a prominent vector of domoic acid poisoning [147]. However, no blooms of potentially dangerous diatoms like *Cerataulina pelagica* or *Pseudo-nitzschia* spp. have as yet been reported from the Pisco Formation [141, 148], and some toxin-producing algae (e.g. *Karenia concordia*) have a non-mineralised skeleton that is unlikely to fossilise. As a result, the role of harmful algal blooms in the context of the Pisco Lagerstätte currently remains speculative.

### Transport and deposition

The large number of specimens preserved ventral-side up suggests that–contrary to previous statements [13]–many specimens floated before sinking to the seafloor (section Disposition). Coincidentally, this may explain high concentrations of large mysticetes (up to 15 m or more in total body length; [149]) in the shallow-water deposits of P0, with floating carcasses accumulating near the coast [35]. Dismemberment during floating led to a loss of completeness, but not necessarily articulation. Instead, disarticulation only became severe following the collapse of the integument after deposition on the seafloor or, at least, after a prolonged period of flotation (see Articulation and completeness). As carcasses sank, they often became reoriented by the prevailing coastal current (see Orientation), but remained relatively untouched by currents, scavengers and benthic invertebrates once on the seafloor (see Shark bite marks, Associated shark teeth, and Associated macro-invertebrates). A few defleshed skeletons remained unburied for long enough to serve as a substrate for encrusting organisms, but most quickly became buried, facilitating both their general preservation and the continued articulation.

### Burial

Background sedimentation rates in the Pisco Formation are insufficient to affect rapid burial, especially of large specimens [35]. Instead, vertebrate carcasses either sank into and became trapped in ‘soupy’ (i.e. waterlogged) sediments like diatomaceous silts; or became covered via scour-induced self-burial (see Inferred mechanisms of rapid burial). Both processes can rapidly cover large specimens, with self-burial offering a particularly promising mechanism for the preservation of substantially articulated skeletons. In addition, soft sediments capable of trapping specimens may be associated with bacterial mats. The latter rarely fossilise [150, 151], but are typical of areas with high productivity and low oxygen levels, and nowadays large coherent microbial communities of the sulphur-oxidising bacterium *Thioploca* occur in the H_2_S rich sediments at 50–280 m depth along the Peruvian and Chilean coast [152–157].

### Oxygen levels at the seafloor

Previous studies on the Pisco Formation suggested than cetacean carcasses did not experience anoxia, given that they are preserved in proximity to tidal and storm-related sedimentary structures [12–14]. Continuous anoxia is further excluded by the presence of non-chemosynthetic infaunal molluscs (e.g. *Dosinia* sp. and *Panopea* sp.) and burrowing traces (e.g. *Gyrolithes vidali* and *Thalassinoides* isp.) at different stratigraphic heights (see Associated macro-invertebrates and Di Celma et al. [19]). On the other hand, some well-preserved vertebrates are found within laminated diatomaceous deposits that are devoid of macro-invertebrates and lack both tractive structures and macroscopical evidence of bioturbation. At the micro-scale, pervasive homogenisation features suggest bioturbation by meiobenthic organisms inhabiting the sediment [141]. Together, these features are consistent with a dysaerobic to quasi-anaerobic facies, and thus dysoxic or even suboxic oxygenation regimes [158]. Whereas decomposition itself might have induced a low-oxygen ’halo’ in the sediment surrounding each decaying carcass [159], oxygen deficiency was–at least at times–likely more widespread at the seafloor as a result of generalised environmental processes and conditions.

Organic matter loading–e.g. via plankton blooms, but see also Walsh [160] and Bakun & Week [161]–could plausibly have led to seasonal shifts from a well-oxygenated sea floor to oxygen-deficient conditions, which in turn may have stifled encrusters and scavengers [162]. Similar fluctuations affect the modern Bay of Paracas (<20 m depth), which is located 70 km north of our study area and one of the most active upwelling zones of the Peruvian coast [163]. During summer, this area experiences chronic hypoxia and anoxic events, accompanied by milky-turquoise waters (‘aguas blancas’) that likely indicate the presence of H_2_S [163]. More generally, the continental shelf of Chile and Peru is well known for its low oxygen concentration on the seafloor, especially around 100–500 m depth [164–168]. Finally, intermittent phases of low oxygenation (oxic-dysoxic to euxinic) have also been proposed for the early Miocene Chilcatay Formation, which directly underlies the Pisco strata [56].

### Mineralisation

Low oxygen levels would have favoured anaerobic decay, which in turn, by locally increasing alkalinity, facilitated the two chief modes of vertebrate preservation characterising the Pisco Lagerstätte: dolomite precipitation and apatite dissolution-recrystallisation (see Bone preservation, Baleen preservation, and Carbonate concretions). Dolomite nodules, sometimes accompanied by phosphatisation, primarily formed around specimens shielded by relatively low-permeability sediment or a dermal skeleton, as well as larger carcasses with enough soft tissue to sustain sulphate reduction (see Baleen preservation and Carbonate concretions). Where nodules formed early, phosphatisation leading to stronger mineralisation but potentially also less preserved detail was limited. Smaller animals like dolphins may fossilise via phosphatisation only, which in the absence of a protective dolomite barrier may continue for a prolonged period [26, 39]. Both dolomite precipitation and phosphatisation may lead to exceptional preservation, including of soft tissues like baleen (see Baleen preservation).

## Concluding remarks

Over the course of 15 field campaigns, we examined and analysed 890 marine vertebrate fossils from the Miocene Pisco Formation of Peru. Their exceptional abundance and preservation qualify this unit as a true Fossil-Lagerstätte, and thus one of the most significant fossiliferous deposits of South America. We use taxonomic, taphonomic, sedimentological and geochemical data to show that there is no single mechanism that can explain the wealth and quality of specimens from the Pisco Formation. Instead, we uncover a complex combination of factors, ranging from original biological abundance to low oxygen levels at the sea floor, rapid burial in a soupy substrate, scour-induced self-burial, and bio-mediated mineralisation, which together created a milieu in which fossilisation was relatively likely. Our results elucidate life and death during the evolution of one of the world’s most productive marine ecosystems (the Humboldt Current), and ultimately may help to explain the formation of other marine vertebrate Fossil-Lagerstätten worldwide.

## Supporting information

S1 Table Taphonomic dataset of fossil marine vertebrates at all the localities in the Ica River Valley. (XLSX)
